# The impact of information and communication technology on immunisation and immunisation programmes in low-income and middle-income countries: a systematic review and meta-analysis

**DOI:** 10.1016/j.ebiom.2024.105520

**Published:** 2024-12-21

**Authors:** Mohini Zarekar, Hussein Al-Shehabi, Rita Dörner, Heide Weishaar, Tessa Lennemann, Charbel El Bcheraoui, Andrea Bernasconi

**Affiliations:** a Evidence-Based Public Health, Centre for International Health Protection, Robert Koch Institute, Berlin, Germany; b Institute of International Health, Charité - Universitätsmedizin Berlin, Berlin, Germany; c Deutsche Gesellschaft für Internationale Zusammenarbeit (GIZ), Bonn, Germany; d Unité Epidémiologie et Recherche Clinique, Réseau de l’Arc, Saint-Imier, Switzerland

**Keywords:** Digital health interventions, Childhood vaccination, Low-income and middle-income countries (LMICs), Health information technology, Vaccine delivery systems, Immunisation equity

## Abstract

**Background:**

Low-income and Middle-income Countries (LMIC) are continually working to ensure everyone can access life-saving vaccines. Recognising the considerable impact of Information and Communication Technology (ICT) in healthcare, we performed a systematic review and meta-analysis to summarise ICT effectiveness in improving vaccine delivery in LMICs.

**Methods:**

A systematic search from January 2010 to August 2023 in MEDLINE, EMBASE, Cochrane Library, BMJ Health & Care Informatics, and grey literature was performed. This search focused on randomised controlled trials (RCTs), non-RCTs, observational, and mixed-methods studies in English, examining ICT's effects on childhood immunisation in LMICs. Risk of bias in RCTs and non-RCTs was assessed using the Joanna Briggs Institute tool, and mixed-methods studies were evaluated with the Mixed Methods Appraisal Tool. A meta-analysis summarised ICT's impact on third pentavalent dose coverage and full immunisation by age one. The study is registered with PROSPERO (CRD42023446062).

**Findings:**

Of 6535 screened studies, 27 involving 354,979 children were included. All apart from one study demonstrated a positive impact on immunisation coverage and timeliness, completeness and accuracy of records, number of adverse events reporting, vaccine stockouts, and cold chain expansion. The meta-analysis demonstrated that reminders effectively improved coverage rate of the third dose of the pentavalent vaccine (OR 2.32, 95% CI 1.34–4.03) and the full immunisation at one year of age (OR 2.61, 95% CI 1.2–5.67) with significant degrees of heterogeneity, respectively I^2^ 82% and I^2^ 89%. Main concerns for bias in RCTs included unblinded outcome assessors and intervention providers. Interpreting quasi-experimental studies was more challenging due to the higher risk of baseline differences between study arms, statistical methods, and dropouts. Mixed-methods studies often lacked clarity in integrating qualitative and quantitative data.

**Interpretation:**

This systematic review confirms the benefits of ICT in immunisation programmes by enhancing various stages of vaccine delivery. Specifically, reminders have been shown to enhance childhood immunisation coverage rates.

**Funding:**

10.13039/501100011099Deutsche Gesellschaft für Internationale Zusammenarbeit (German Corporation for International Cooperation, GIZ) as part of the Digital Innovation in Pandemic Control (DIPC) Initiative, financed by the Bundesministerium für Wirtschaftliche Zusammenarbeit (Federal Ministry for Economic Cooperation and Development, BMZ).

**Research in context**

**Evidence before this study**

The COVID-19 pandemic has served as a major catalyst for the adoption and integration of Information and Communication Technologies (ICT) in healthcare, particularly in the context of vaccine distribution. This unprecedented global health crisis has necessitated innovative approaches to ensure efficient and equitable vaccine delivery. Consequently, we were keen to investigate how this surge in ICT utilisation has transformed vaccine delivery systems in Low-income and Middle-Income Countries (LMIC) in comparison to the past. Thus, we looked for studies published from January 2010 up to August 2023 to assess the effectiveness of these technologies in overcoming logistical challenges, enhancing data management for immunisation programmes, and improving access to vaccines for remote and underserved populations. We hypothesised that the increasing penetration of mobile phone usage in LMICs presents an important opportunity to leverage ICT for more efficient vaccine delivery.

**Added value of this study**

This systematic review and meta-analysis assesses the impact of ICT across all stages of vaccine delivery in LMICs, encompassing aspects from stock management to the actual administration of vaccines. It offers a comprehensive analysis of how ICT can streamline and enhance the entire vaccine supply chain, providing valuable insights into its effectiveness in improving healthcare outcomes.

**Implications of all the available evidence**

Our study provides robust evidence confirming the considerable potential of ICT in enhancing vaccine delivery systems in LMICs. Through our comprehensive analysis, we have identified several ways in which ICT can revolutionise this process: from streamlining logistics and supply chain management to ensuring more efficient and timely administration of vaccines. Our meta-analysis, which focused on the effect of vaccination-slot reminders on full immunisation rates at one year of age (across five studies) and on DPT-3/Penta-3 coverage rates (across seven studies), showed odds ratios (ORs) of 2.6 and 2.3, respectively, indicating increased odds of coverage.

Nonetheless, it is important to note that we observed considerable heterogeneity among the studies, with an I^2^ value exceeding 80%. Furthermore, our evaluation of digital interventions highlighted several critical shortcomings, including limited interoperability, a lack of cost-effectiveness analyses, and inadequate focus on data protection and the adoption of Digital Public Goods. Additionally, we found no evidence of effectiveness of any solutions being specifically implemented during the COVID-19 pandemic. From this analysis, we are able to provide valuable recommendations for future research and actionable strategies for developers as our assessment highlight the current limitations in both methodology and study reporting, which must be overcome to make further improvements in this field.

## Introduction

Annually, vaccine-preventable diseases cause 1.5 million fatalities among children under five, mostly in Low-income and Middle-Income Countries (LMIC).^1^ Recognised as a vital public health intervention,^2^ the World Health Organization (WHO) introduced the Expanded Programme on Immunisation (EPI) in 1974, marking the beginning of a concerted international effort to use immunisation as a fundamental public health strategy.^3^ The EPI substantially reduced child mortality and morbidity from diseases like measles and polio. Vaccines administered via the EPI prevent 2.5 million deaths annually^4^ and it is estimated that vaccinations against ten key pathogens could avert approximately 69 million deaths from 2000 to 2030.^5^

However, despite continuous efforts, approximately 21 million children worldwide remained either unvaccinated or under-vaccinated in 2023. The first-dose measles vaccination rate decreased from 86% in 2019 to 83%, and approximately 84% of infants worldwide (108 million) received three doses of the DTP3 vaccine. These coverages are far from sufficient to prevent the onset of epidemics, and furthermore, these global figure mask substantial disparities, with LMICs lagging behind.^6^ The WHO State of Inequalities in Childhood Immunization Report, which is based on data from 67 LMICs, indicates that the interquartile range for full vaccination coverage was 51%–83%, reflecting substantial inequities in socio-economic status and health service access across different regions.^7^

The COVID-19 pandemic has further weakened immunisation programmes. The pandemic disrupted vaccine supply chains,^8^^,^^9^ further accentuated the disparities between high-income and LMICs and underscoring the need for stronger immunisation strategies in the latter.^10^ To bolster immunisation programmes, the Immunization Agenda 2030^10^ encourages the use of information and communication technology (ICT).^2^ During the COVID-19 pandemic, most of the high-income countries and LMICs adopted at least one ICT solutions to track immunisations, establish vaccination records, issue digital certificates, and report side effects.^11^, ^12^, ^13^ In LMICs, ICT is expected to aid in reaching vulnerable populations, thereby supporting the achievement of the Sustainable Development Goals and Universal Health Coverage.^10^ GAVI, the Vaccine Alliance, advocates ICT adoption,^14^ leveraging the increasing mobile phone penetration in LMICs. In fact, of the seven billion mobile phone users worldwide, 70% reside in LMICs,^15^ and it is estimated that in Sub-Saharan Africa, mobile broadband will constitute 87% of mobile connections by 2025, which would be a substantial increase from the 2018 figure of 38%.^16^

The role of digital technologies in clinical medicine is well-documented,^17^^,^^18^ but their impact on public health in LMICs, particularly for disease prevention, is less clearly defined. Evaluating ICT solutions in line with current guidelines and needs is also crucial, as technology evolves rapidly and may become obsolete, potentially failing to meet contemporary challenges. Additionally, SAGE recommendations for updating guidelines often establish new standards important for technical, legal and even ethical compliance.^19^ Acknowledging research gaps in digital health and vaccination, WHO advocates for additional research and guidance^20^ to achieve SDGs by reducing vaccine-preventable diseases and expanding access to new vaccines before 2030.^2^

Our systematic review and meta-analysis aim to evaluate the impact and effectiveness of ICT on immunisation programmes. This includes exploring to what extent ICT interventions can enhance vaccine delivery, improve data management, and increase patient outreach and engagement.

## Methods

### Ethics

No ethical approval was sought for the conduct of this systematic review and meta-analysis, because no primary data collection involving human or animal subjects took place, and the data and analyses presented are based on previously published peer-reviewed research.

### Search strategy and selection criteria

This systematic review and meta-analysis followed the Preferred Reporting Items for Systematic Reviews and Meta-Analyses (PRISMA)^21^ guidelines and its protocol is registered with PROSPERO (CRD42023446062). Focusing on English-language, peer-reviewed studies, we evaluated ICT interventions in immunisation programmes within LMICs, involving healthcare workers (HWs), caregivers, or health managers.

We assessed ICT interventions like electronic health records, mHealth, and mobile apps, comparing their effect on immunisation services with traditional or non-ICT approaches. We considered any effects on immunisation coverage rates, dropout rates, timeliness and accuracy of reporting, vaccine stock-outs, cold chain management, and Adverse Events Following Immunisation (AEFI) rate. Our study included RCTs, quasi-experimental, observational, and mixed-method designs.

Our search was conducted using the databases MEDLINE, EMBASE, Cochrane Library, and BMJ Health & Care Informatics. Studies published from January 2010 to August 2023 were included to capture the considerable growth in digital health and telehealth research over the past decade, particularly following the COVID-19 pandemic,^22^ as well as to reflect the evolving landscape of digital health since the establishment of the ‘Principles for Digital Development’ in 2010.^23^ We screened for relevant literature, including grey literature, using specific keywords and Boolean operators. For grey literature we followed the AACODS (Authority, Accuracy, Coverage, Objectivity, Date, Significance) checklist.^24^

Two authors (MZ and AB) independently screened studies, resolving disagreements through discussion or a third reviewer (HAS). For each study that met the inclusion criteria, we retrieved and analysed the full text, extracting key details like study design, objective, implementation country, digital intervention type, and outcomes. The same authors independently assessed the quality of the studies. RCTs, non-RCTs, and quasi-experimental studies were appraised using Joanna Briggs Institute's 2023 tools,^25^ while mixed-method studies used the 2018 Mixed Methods Appraisal tool.^26^ Studies were rated as high, medium, or low risk of bias, to identify potential shortcomings that could impact their conclusions. Due to the impracticality of blinding in digital interventions, aspects of the assessment tools related to blinding were disregarded.

### Risk of bias assessment

We used the 2023 Joanna Briggs Institute critical appraisal tools for RCTs, quasi-experimental, and cross-sectional studies^25^ to evaluate studies according to their relative design. In the case of mixed-methods studies, we used the Mixed Methods Appraisal Tool, 2018 from Quan Nha et al.^26^ Further, to assess potential publication bias, we generated funnel plots showing the distribution of Odds Ratios of third dose pentavalent vaccine and full immunisation at one year of age against Standard Errors from the studies used in the meta-analysis (Supplementary File 1). We did not perform a statistical test for funnel plot asymmetry due to the limited number of studies included in the meta-analysis and the significant heterogeneity between them.^27^

### Statistics

A meta-analysis of RCTs evaluated the impact of ICT on two key immunisation indicators: full vaccination coverage by age one and the coverage rate of the third dose of the pentavalent vaccine (Penta-3).

The Pentavalent vaccine covers Diphtheria, Pertussis, Tetanus, Hepatitis B and *Haemophilus influenzae* type b (Hib) and Penta 3 coverage is the percentage of children who have received three doses of the pentavalent vaccine. We chose pentavalent third dose coverage as one of the most important indicators in measuring immunisation coverage and a common indicator used in all RCTs, in terms of dose frequency and timing.^28^ Full vaccination by age one was defined as the proportion of one-year-olds who received all recommended vaccines according to national guidelines.^29^ We focused on intention-to-treat results to minimise bias and reflect real-world conditions in LMICs. For studies with multiple arms sharing a common control group, we aggregated the results by calculating a weighted average of the effects, rather than analysing the arms separately. Finally, we reported the degree of inter-study heterogeneity using the Cochrane's Q test. Where a significant heterogeneity was present (p ≤ 0.10 or I^2^ >50%) we applied a random-effects model, with further subgroup analysis for specific effects. R, version 4.3.1, and the ‘meta’ package were used to conduct this analysis.

We classified the outcomes of the studies included in our review into two tiers based on their focus: population-level and system-level outcomes. Population-level outcomes referred to those that determine the completeness, coverage, and timeliness of immunisations. The benefits in this case directly pertain to the target population. System-level outcomes improve the efficiency of the immunisation programme itself, either through the implementation of new electronic health record systems or by maintaining an effective supply chain.

PICOS framework and research strategy are presented in Supplementary File 2.

### Role of funders

The funders had no role in the study design, methodology, literature search, or data analysis. However, TL, who is an employee of the funding agency and co-author on this paper, contributed technical expertise during the writing, reviewing and editing process. Her input reflects her personal views and not those of the funder.

## Results

**Fig. 1:**
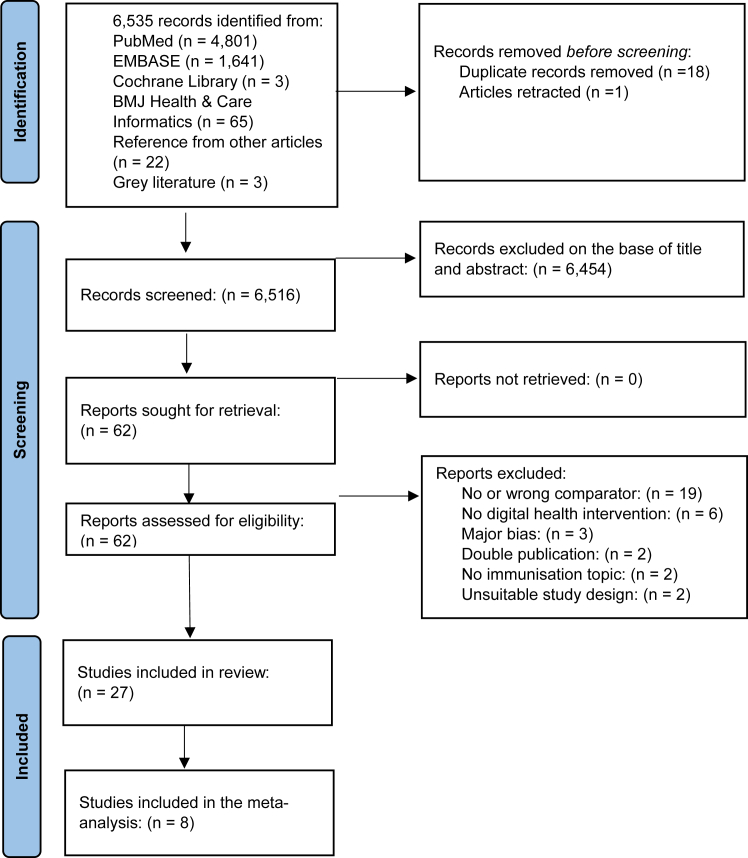
PRISMA (Preferred Reporting Items for Systematic Reviews and Meta-Analyses) flow diagram of the studies selection process^1^. PRISMA flow diagram outlining the study selection process. A total of 6535 records were identified through database searches and other sources. After the removal of duplicates and screening, 62 full-text articles were assessed for eligibility, resulting in the inclusion of 27 studies in the systematic review, 8 of which were included in the meta-analysis. Reasons for exclusion at each stage are provided. ^1^Excluded reports: Major bias (n = 3): One study experienced an implementation failure unrelated to the ICT intervention, therefore its impact could not be assessed. Two studies had significant loss to follow-up, which introduced a high risk of bias in their findings. Unsuitable study design (n = 2): One study provided only the study protocol without any results, and another focused solely on the development of the intervention, rather than its implementation or evaluation.

Our initial search across medical databases and grey literature yielded 6535 studies. Upon removing 18 duplicates and one retracted article, and excluding 6414 based on title and abstract for not meeting our criteria, we retrieved full texts for the remaining 62 articles. After an in-depth review, 35 further articles were excluded, leaving 27 studies for our analysis. Studies that were considered for inclusion but rejected are described in Supplementary File 3. The PRISMA flow diagram is shown in Fig. 1.

### Characteristics of included studies

Of the 27 studies included in our analysis, 15 were RCTs,^30^, ^31^, ^32^, ^33^, ^34^, ^35^, ^36^, ^37^, ^38^, ^39^, ^40^, ^41^, ^42^, ^43^, ^44^ seven were quasi-experimental,^45^, ^46^, ^47^, ^48^, ^49^, ^50^, ^51^ four used mixed methods,^52^, ^53^, ^54^, ^55^ and one was an observational study.^56^ These studies, conducted in 17 LMICs, mainly in Africa (20 studies) and Asia (6 studies), with one in South America, targeted a total of 354,979 children. Key outcomes reported included childhood immunisation coverage and timeliness (20 studies),^30^, ^31^, ^32^^,^^34^, ^35^, ^36^, ^37^^,^^39^, ^40^, ^41^, ^42^, ^43^, ^44^, ^45^, ^46^, ^47^, ^48^, ^49^, ^50^^,^^54^ data completeness and accuracy (3 studies),^52^^,^^53^^,^^56^ supply chain improvement (3 studies)^45^^,^^51^^,^^55^ and AEFI (2 studies).^33^^,^^38^

ICT interventions predominantly involved digital reminders (19 studies), including automatic and manual Short Message System (SMS) and phone calls^4^^,^^30^, ^31^, ^32^, ^33^, ^34^, ^35^, ^36^, ^37^^,^^39^, ^40^, ^41^, ^42^, ^43^, ^44^^,^^46^^,^^48^^,^^49^^,^^54^ primarily for vaccination appointment reminders and AEFI alerts. Other interventions included electronic immunisation registries (EIR) (3 studies),^47^^,^^52^^,^^56^ software tools (3 studies)^39^^,^^43^^,^^53^ or other digital solution (1 study) for vaccine stock and cold chain management.^45^^,^^51^^,^^55^ Detailed characteristics of these studies are in Supplementary Files 4 (Summary of included studies) and Supplementary Files 5 (Intervention description).

### Risk of bias

The risk of bias assessment identified 15 studies (55.6%) with a low risk of bias, eight (29.6%) with a medium risk, and four (14.8%) with a high risk. The detailed bias assessment is presented in Supplementary File 6. Major concerns in RCTs included non-blinding of outcome assessors (73.3%) and treatment providers (53.3%). Quasi-experimental studies mainly faced issues with differences between intervention and control arm (77.8%), statistical analysis (77.8%), and loss to follow-up (66.7%). In about half of the mixed-methods studies we reviewed; it was difficult to discern how the results from the qualitative methods were connected to the results from the quantitative methods. When we assessed the risk of publication bias, the plots exhibited some degree of asymmetry, indicating that smaller studies, such as Haji (2016) and Bangure (2015), which reported null or negative results, may be underrepresented.

### Population-level outcomes

Nineteen studies (70%) focused on reminder-based interventions for scheduled vaccination appointments. Digital reminders generally improved immunisation coverage rates compared to control groups. Eight studies^31^^,^^35^, ^36^, ^37^^,^^39^^,^^40^^,^^43^^,^^44^ noted enhanced uptake of Penta-3; four reported timeliness^34^^,^^39^^,^^47^^,^^49^ in immunisation, others reduced dropout rates^37^ and higher return rates to facilities.^36^^,^^40^ Two studies^34^^,^^35^ noted that combining reminders with monetary incentives synergistically increased vaccination rates, and another showed increased uptake when reminders were combined with EPI training in primary healthcare.^54^ One study from Vietnam investigated parental willingness to pay out-of-pocket for an ICT solution, as an outcome, using a behavioural survey, and findings indicated positive attitudes in this regard.^49^ Another study examined return rates for vaccinations, observing an increase due to the CIMA^50^ app. A summary of the coverage attained through reminders is presented in Table 1.

**Table 1:** Comparison of immunisation coverage rates attained under Information and Communication Technology and usual care.

Studies	Vaccine	ICT	UC	Effect size (95% CI)	p-value	Intervention
Bangure et al. (2015)^31^	PCV-1	96.7%	82.2%	OR 6.3 (2.4–16.9)	<0.01	Reminders only
Bangure et al. (2015)^31^	PCV-2	96.1%	80.3%	OR 5.9 (2.4–14.8)	<0.01	Reminders only
Bangure et al. (2015)^31^	PCV-3	94.7%	71.7%	OR 7.1 (3.2–15.7)	<0.01	Reminders only
Bangure et al. (2015)^31^	Penta-1	96.7%	82.2%	OR 6.3 (2.4–17.0)	<0.01	Reminders only
Bangure et al. (2015)^31^	Penta-2	96.1%	80.3%	OR 6.0 (2.4–14.8)	<0.01	Reminders only
Bangure et al. (2015)^31^	Penta-3	94.7%	71.7%	OR 7.1 (3.2–15.7)	<0.01	Reminders only
Bangure et al. (2015)^31^	Polio-1	96.7%	82.2%	OR 6.3 (2.4–17.0)	<0.01	Reminders only
Bangure et al. (2015)^31^	Polio-2	96.1%	80.3%	OR 6.0 (2.4–14.8)	<0.01	Reminders only
Bangure et al. (2015)^31^	Polio-3	94.7%	71.7%	OR 7.1 (3.2–15.7)	<0.01	Reminders only
Dissieka et al. (2019)^36^	DPT-1	86.6%	76.1%	aOR 2.8 (1.8–4.3)	<0.01	Reminders only
Dissieka et al. (2019)^36^	DPT-2	81.0%	67.3%	aOR 2.8 (1.9–4.2)	<0.01	Reminders only
Dissieka et al. (2019)^36^	DPT-3	74.2%	58.3%	aOR 2.7 (1.8–3.9)	<0.01	Reminders only
Dissieka et al. (2019)^36^	MMR^a^	60.7%	37.8%	aOR 4.5 (2.8–7.2)	<0.01	Reminders only
Ekhaguere et al. (2019)^39^	Measles	73.3%	65.3%	OR 1.5 (1.0–2.1)	<0.05	Reminders only
Ekhaguere et al. (2019)^39^	Penta-1	95.0%	96.3%	OR 1.2 (0.8–1.9)	0.43	Reminders only
Ekhaguere et al. (2019)^39^	Penta-2	92.0%	92.7%	OR 1.1 (0.7–1.7)	0.65	Reminders only
Ekhaguere et al. (2019)^39^	Penta-3	85.7%	81.3%	OR 1.4 (0.9–2.1)	0.15	Reminders only
Gibson et al. (2017)^35^	Measles	87.1%	83.9%	OR 1.0 (0.9–1.1)	0.21	Reminders only
Gibson et al. (2017)^35^	Penta-1	99.7%	99.7%	OR 1.0 (0.9–1.2)	0.96	Reminders only
Gibson et al. (2017)^35^	Penta-2	98.7%	98.9%	OR 1.0 (0.9–1.1)	0.82	Reminders only
Gibson et al. (2017)^35^	Penta-3	96.6%	98.1%	OR 0.9 (0.9–1.1)	0.24	Reminders only
Gibson et al. (2017)^35^	Polio-1	99.5%	99.7%	OR 1.0 (0.9–1.1)	0.61	Reminders only
Gibson et al. (2017)^35^	Polio-2	98.7%	98.6%	OR 1.0 (0.1-1.2)	0.9	Reminders only
Gibson et al. (2017)^35^	Polio-3	95.9%	96.9%	OR 1.0 (0.9–1.0)	0.44	Reminders only
Haji et al. (2016)^37^	Penta-2	98.1%	91.4%	OR 4.9 (2.1–11.3)	0.42	Reminders only
Haji et al. (2016)^37^	Penta-3	96.5%	83.1%	OR 5.6 (3.0–10.4)	0.31	Reminders only
Kagucia et al. (2021)a^32^	MCV-1	78.1%	68.1%	OR 1.7 (1.1–2.8)	0.05	Reminders only
Kagucia et al. (2021)b^32^	MCV-1	77.8%	68.1%	OR 1.6 (0.9–2.7)	0.06	Reminders + 1USD
Kagucia et al. (2021)a^32^	MCV-2	84.2%	78.1%	OR 1.5 (0.8–2.7)	0.17	Reminders only
Kagucia et al. (2021)b^32^	MCV-2	84.6%	78.1%	OR 1.5 (0.9–2.7)	0.15	Reminders + 1USD
Kazi et al. (2018)^44^	Penta-1	76.0%	71.3%	OR 1.3 (0.8–2.1)	0.36	Reminders only
Kazi et al. (2018)^44^	Penta-2	58.7%	52.7%	OR 1.3 (0.8–2.0)	0.30	Reminders only
Kazi et al. (2018)^44^	Penta-3	31.3%	26.0%	OR 1.3 (0.8–2.1)	0.31	Reminders only
Mekonnen et al. (2021)^40^	Measles	91.5%	79.3%	OR 2.8 (1.6–5.1)	<0.01	Reminders only
Mekonnen et al. (2021)^40^	Penta-1	98.6%	95.3%	OR 3.4 (0.9–12.7)	0.06	Reminders only
Mekonnen et al. (2021)^40^	Penta-2	98.1%	90.6%	OR 5.4 (1.8–16.1)	<0.01	Reminders only
Mekonnen et al. (2021)^40^	Penta-3	95.8%	86.9%	OR 3.4 (1.6–7.5)	<0.01	Reminders only
Yunusa et al. (2022)^48^	Penta-1	77.4%	71.9%	OR 1.3 (0.9–2.0)	0.13	Reminders only
Yunusa et al. (2022)^48^	Penta-2	68.3%	48.9%	OR 2.2 (1.6–3.2)	<0.01	Reminders only
Yunusa et al. (2022)^48^	Penta-3	60.1%	43.3%	OR 1.97 (1.4–2.8)	<0.01	Reminders only

OR, Odds Ratio.

aOR, Adjusted Odds Ratio.

DPT, Diphtheria, Pertussis, and Tetanus (3 doses).

IPV, Inactivated Poliovirus Vaccine.

Penta, Diphtheria, Pertussis, Tetanus, Hepatitis B, and *Hemophilus influenzae* type b (3 doses).

MCV, Measles containing vaccine (2 doses).

MMR, Measles, Mumps, and Rubella vaccine.

PCV, Pneumococcal Conjugate Vaccine.

UC, Usual Care.

YF, Yellow Fever.

CI, Confidence Interval.

ICT, Information and Communication Technology.

a For Dissieka et al. (2019): Please note that there was a minor error regarding the vaccine type. In Côte d'Ivoire, the measles-rubella (MR) vaccine is used, rather than the MMR vaccine.

**Fig. 2:**
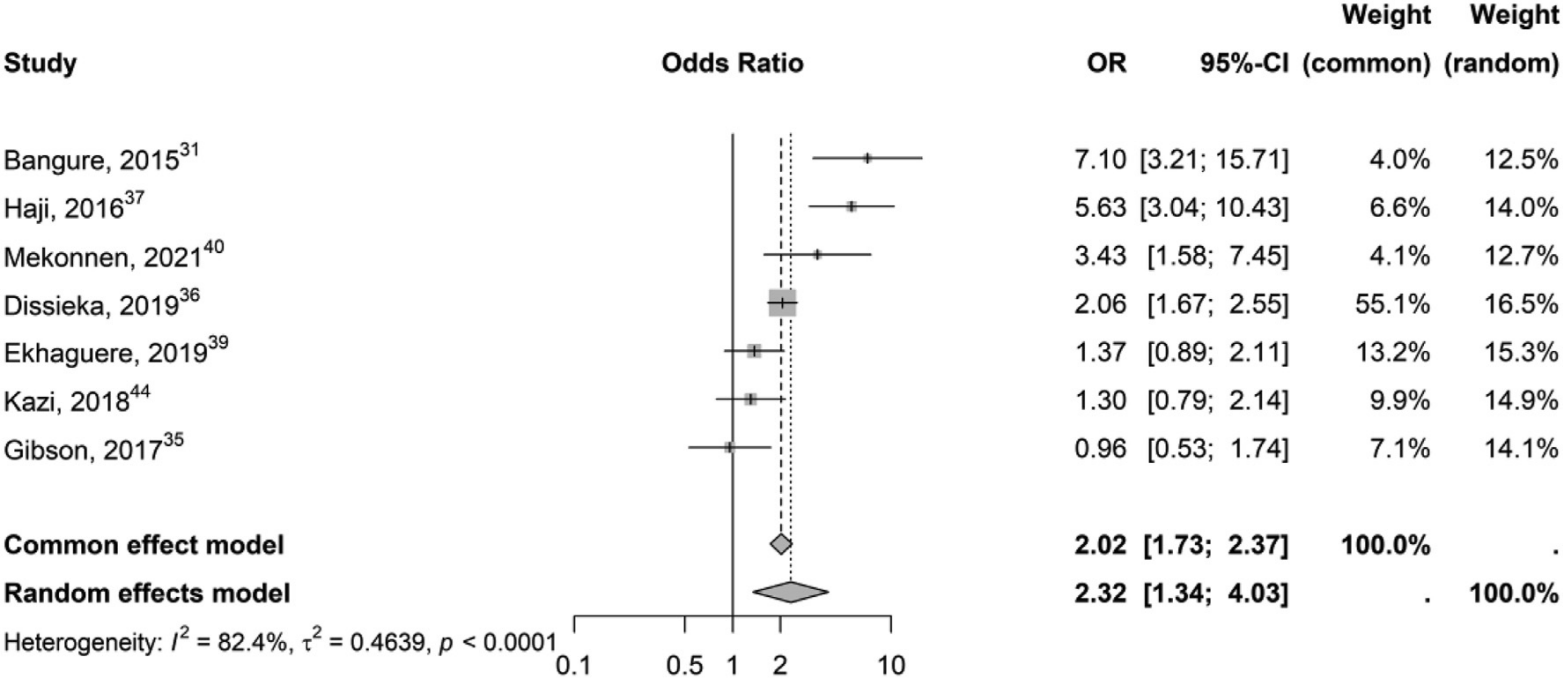
Forest plot presenting pooled odds ratios with 95% confidence intervals for studies assessing the impact of ICT interventions on the coverage rate of the third dose of the pentavalent vaccine (Penta-3). Study-specific and overall effect sizes are shown, with heterogeneity among studies quantified by the I^2^ statistic.

The meta-analysis on Penta-3 coverage included seven RCTs^31^^,^^35^, ^36^, ^37^^,^^39^^,^^40^^,^^44^ involving 6290 children. Six interventions^35^ reported increased vaccination coverage in the intervention group, with four showing statistically significant improvements.^31^^,^^36^^,^^37^^,^^40^ The pooled odds ratio was 2.3 (95% CI 1.3–4.0) with heterogeneity high amongst the interventions (I^2^ = 82.4%) (Fig. 2).

The subgroup analysis revealed varying effectiveness based on user focus (higher for caregiver-focused interventions), study design (stronger in RCTs), sample size (greater in smaller studies), and study quality (better results in lower-quality studies) (Table 2).

**Table 2:** Subgroup analysis of the effectiveness of SMS reminders on improving coverage of the third dose of the pentavalent vaccine.

Study categories	Number of studies	OR	95% CI	I^2^^a^
All	7	2.3	(1.3–4.0)	82.4
High quality study	5	2	(1.0–4.0)	81.1
Low quality study	2	3.3	(1.2–8.7)	89.1
Caregivers as users of the intervention	5	2.3	(1.3–4.1)	77.1
HWs as users of the intervention	2	2.3	(0.4–13.1)	93.9
RCT study design	6	2.7	(1.5–4.7)	81.9
cRCT^b^ study design	1	1	(0.5–1.7)	NA
Small sample size (≤300)	3	3	(1.1–8.1)	85.4
Big sample size (>300)	4	1.9	(1.0–4.0)	84.7
Continent–Africa	6	2.6	(1.4–4.8)	83.8
Continent–Asia	1	1.3	(0.9–2.1)	NA

OR, Odds ratio.

95% CI, 95% Confidence Interval.

HW, Healthcare workers.

RCT, Randomised controlled trial.

cRCT, Cluster randomised controlled trial.

a I^2^: Statistical heterogeneity.

b For Dissieka et al. (2019): Please note that there was a minor error regarding the vaccine type. In Côte d'Ivoire, the measles-rubella (MR) vaccine is used, rather than the MMR vaccine.

All studies^35^^,^^36^^,^^39^, ^40^, ^41^ (n = 5) reported significant improvements in full vaccination coverage at one year of age for a total of 5859 children involved (Table 3).

**Table 3:** Comparison of full immunisation coverage rate at one year of age under the digital health intervention and usual care.

Study	Digital health	Usual care	Effect size (95% CI)	p-value	Additional intervention
Brown et al. (2016)a^41^	98.7%	74.7%	OR 25.1 (5.9–106.3)	<0.01	Reminders only
Brown et al. (2016)a^41^	98.6%	74.7%	OR 12.1 (4.2–35.0)	<0.01	Reminders only + specific training
Dissieka et al. (2019)^36^	58.3%	35.7%	OR 2.5 (2.1–3.1)	<0.01	Including Vitamin A administration
Gibson et al. (2017)a^35^	99.4%	82.2%	OR 1.3 (0.9–1.9)	0.21	Reminder only
Gibson et al. (2017)b^35^	99.5%	82.2%	OR 1.3 (0.9–1.9)	0.16	Reminder + 0.5USD
Gibson et al. (2017)c^35^	99.5%	82.2%	OR 1.9 (1.2–2.9)	<0.01	Reminder + 1.3USD
Ekhaguere et al. (2019)^39^	74.0%	66.0%	RR 1.1 (1.0–1.25)	0.03	–
Mekonnen et al. (2021)^40^	82.6%	70.9%	OR 2.0 (1.2–3.1)	<0.01	–

aRR, Adjusted Risk Ratio.

RR, Relative Risk.

CI, Confidence interval.

**Fig. 3:**
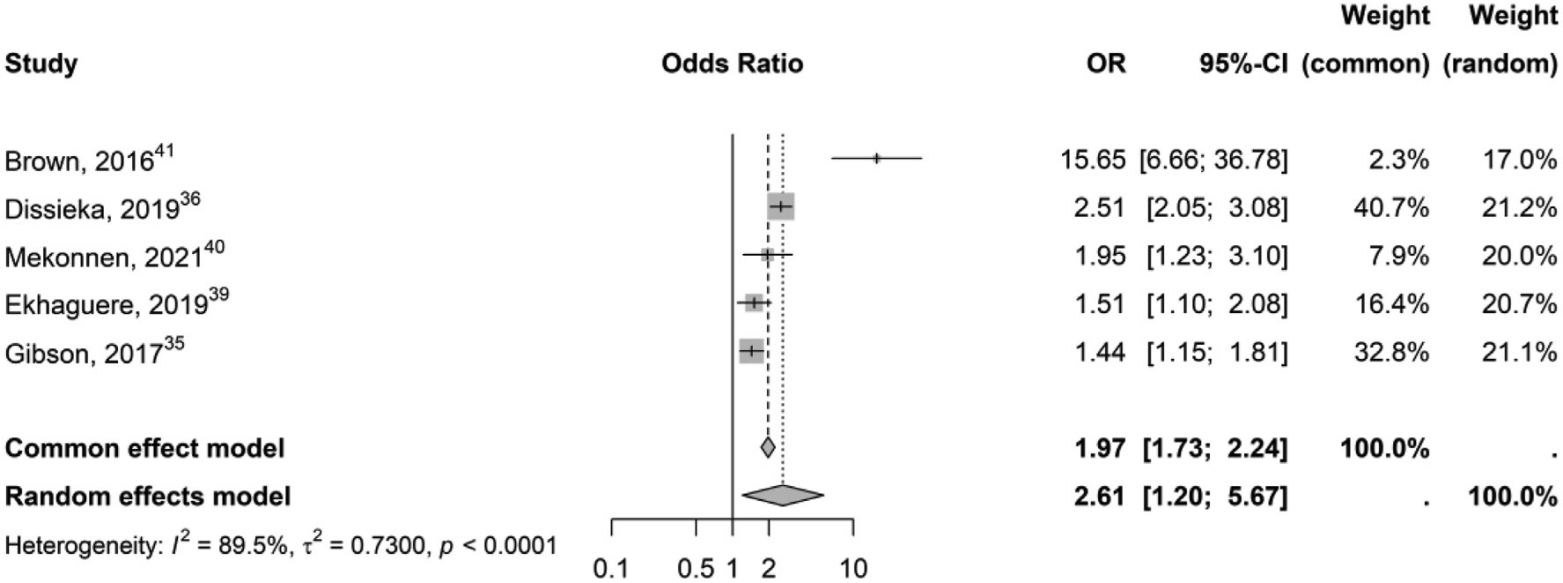
Forest plot showing pooled odds ratios with 95% confidence intervals for studies evaluating the impact of ICT interventions on achieving full immunisation by one year of age. Heterogeneity across studies is indicated by the I^2^ statistic.

The pooled OR was 2.6 (95% CI 1.4-4.8) with a high level of heterogeneity (I^2^ = 89.1%) (Fig. 3).

The subgroup analysis showed variation in effect size based on target population (higher effectiveness for caregiver-targeted interventions), research quality (larger effects in higher-quality studies), and sample size (greater OR in studies with over 300 participants) (Table 4).

**Table 4:** Subgroup analysis of the effectiveness of SMS reminders on the coverage of full immunization at one year of age.

Study categories	Number of studies	OR	95% CI	I^2^^a^
All	5	2.6	(1.2–5.7)	89.1
High quality study	3	1.5	(1.3–1.8)	0
Low quality study	2	6	(1.0–35.8)	94
Caregivers as users of the intervention	3	2	(1.4–2.7)	71.6
HWs as users of the intervention	2	4.6	(0.4–47.3)	96.4
RCT study design	4	3.1	(1.2–8.1)	90
cRCT^b^ study design	1	1.4	(1.1–1.8)	NA
Small sample size (≤300)	2	5.3	(0.7–41.1)	94.3
Big sample size (>300)	3	1.8	(1.2–2.6)	86.5

OR, Odds ratio.

95% CI, 95% Confidence Interval.

HW, Healthcare workers.

RCT, Randomised controlled trial.

cRCT, Cluster randomised controlled trial.

a I^2^: Statistical heterogeneity.

b For Dissieka et al. (2019): Please note that there was a minor error regarding the vaccine type. In Côte d'Ivoire, the measles-rubella (MR) vaccine is used, rather than the MMR vaccine.

### System level outcomes

Two studies assessed AEFI reporting by caregivers: one using a “beep” alert^33^ and the other through standardised SMS.^38^ Both studies noted increased reporting rates, but statistical significance was only observed in the study utilising “beep” alerts.

Two more studies^45^^,^^55^ evaluated Effective Vaccine Management (EVM) tools, which digitise the immunisation supply chain for better vaccine delivery, storage, and management. Endorsed by WHO, EVM helps optimise resource allocation and supply chain efficiency.^57^ One Indian study^55^ showed an overall 2.8% improvement in vaccine cold storage space utilisation, with notable improvements at district and regional levels. Another study in Benin and Mozambique^45^ found significant increases in cold chain capacity (from 40% to 100%, Mozambique) and reduced stockouts (from 79% to 1%, Benin), leading to an increase in DTP-3 vaccine coverage (from 68.9% to 92.8%).

Three studies^47^^,^^52^^,^^56^ evaluated EIR for data accuracy compared to traditional paper methods. One study assessed VaxTrac immunisation registry,^52^ finding 86% data alignment with paper records in larger facilities but only 43% in smaller ones. In Mongolia, a national EIR showed over 90% congruence with paper data and improved completeness by 78.9%. However, the accuracy of this EIR declined over time. In Tanzania, an EIR was introduced as part of a package including a tablet-based software for automated and simplified reports, a logistics management information system, and a WhatsApp support network. While initially on-time vaccination rates dropped, detailed analysis indicated improved timing for fully recorded vaccinations.^47^

## Discussion

This systematic review indicates that ICT can significantly enhance immunisation programmes in LMICs. By focusing on LMICs, this review addresses immunisation challenges within populations that often face systemic healthcare access barriers, thereby including marginalised groups in the assessment of digital health interventions. Among the 27 studies reviewed, 19 showed that ICT, particularly through reminders, improves vaccination rates and timeliness.^30^, ^31^, ^32^, ^33^, ^34^, ^35^, ^36^, ^37^^,^^39^, ^40^, ^41^, ^42^, ^43^, ^44^^,^^46^^,^^48^^,^^49^^,^^54^ One study^33^ indicated better AEFI reporting, and three studies^47^^,^^52^^,^^56^ highlighted improvements in immunisation registries' data accuracy and completeness. Additionally, three studies^45^^,^^51^^,^^55^ suggested that ICT enhance supply chain management.

Our meta-analysis, examining the effect of reminders on full immunisation coverage at one year and DPT-3/Penta-3 coverage, revealed odds ratios (ORs) of 2.3 and 2.6, respectively, indicating increased odds of coverage. However, significant heterogeneity was observed, likely due to differences in interventions, methodologies, and population characteristics. Subgroup analysis revealed that incentives and caregiver-targeted solutions yielded better outcomes for DPT-3/Penta-3 coverage, while larger, high-quality studies without incentives showed more improvement in full coverage. These findings align with similar studies^4^^,^^58^ and the effectiveness of reminders was already observed in other medical contexts, including medication adherence,^59^, ^60^, ^61^ clinic appointment attendance,^62^^,^^63^ and participation in antenatal care programmes.^62^, ^63^, ^64^ However, the use of reminders mainly focused only on the administration aspect of vaccine delivery, often as standalone solutions. Only two studies^33^^,^^49^ mentioned data synchronisation with EPI, antenatal care, and family planning. Despite the importance of interoperability in health systems for data sharing and interpretation,^65^^,^^66^ our review found no comprehensive digital ‘suites’. Integrated software applications (named “suite”) can enhance management by merging immunisation data with procurement and broader health intervention data, offering real-time supply insights and aiding in outbreak management.^67^ The data suggests that, combining multiple digital reminders delivered through multiple platforms is more effective than a single email reminder in high-income countries.^68^ The lack of interoperability with electronic medical records may result also in missed immunisation opportunities.^69^ However, the COVID-19 pandemic has spurred a rapid increase in digital tool adoption, advancing technological integration by several years.^70^ Despite low digital maturity in many LMICs,^70^^,^^71^ more and more governments are adopting digital health strategies,^72^, ^73^, ^74^ and Africa is seeing a rise in digital entrepreneurship. By 2025, expanded mobile connections could facilitate a transition to ICT solutions.^75^ Yet, challenges like handset affordability, and gender and geographical disparities in mobile internet use, remain. For instance, the costliest handsets consume 54% of the poorest population's monthly income, rural adults are 33% less likely to use mobile internet than urban counterparts, and women are 16% less likely than men to use it.^76^ While reminders are a prevalent ICT tool for enhancing immunisation programmes, their effectiveness may be limited in less advantaged groups, especially those without personal mobile phones or charging means.^33^ The success of reminders depends on caregivers' technological literacy and capacity to understand written messages. Hence, implementing electronic reminders or similar interventions could inadvertently exacerbate inequalities related to education and wealth, especially among groups or in areas where digital literacy levels are low.^77^ One study confirmed this notion, as caregivers preferred receiving phone calls in their local language over digital reminders, pointing towards accessibility issues, but also towards barriers to using technological solutions compared to traditional means of communication.^33^ Additionally, limited knowledge about vaccines can be a challenge, as children of mothers with less education may be less likely to receive vaccinations.^77^

In our research, we encountered an additional noteworthy finding. The COVID-19 pandemic jeopardised routine vaccinations in 68 countries, affecting over 80 million children worldwide due to supply chain disruptions, reduced health workforce, and resource reallocation.^8^^,^^9^^,^^78^ Contrary to the recommendations given by the WHO, GAVI, the vaccine alliance and the World Bank^79^ to integrate and scale up COVID-19 vaccination into EPIs, the new vaccine campaign often competed for resources like transport, cold chain logistic and human resources.^8^^,^^80^ Nonetheless, we identified 26 digital tools^12^ and some commendable examples from upper-middle-income countries like India and Indonesia.^81^^,^^82^ yet this review did not find any trials evaluating their efficacy or that of any other tools employed in the COVID-19 campaign in LMICs. This lack of evidence in scientific literature, especially during a period of accelerated digital healthcare transformation, suggests either a gap in documentation or a focus on immediate tool deployment over detailed efficacy research. However, the pandemic fostered an important dialogue around ICT use in public health initiatives.^80^

Indeed, and despite global immunisation coverage having flatlined^83^ and jeopardised during the pandemic, ICT offers considerable potential to boost vaccine uptake, aligning with WHO's Immunisation^83^ Agenda 2030 goals.^2^ Mobile phone access in LMICs often surpasses basic utilities like electricity, clean water or adequate sanitation.^84^ Telemedicine has improved healthcare accessibility in remote areas,^85^^,^^86^ and the shift from paper to electronic health records has increased efficiency and accuracy in patient data management.^87^ Many LMICs have already initiated the use of data tools and information systems to monitor zero-dose children.^88^ The future promises further advancements with AI and Machine Learning in diagnostics.^89^

However, many reviewed studies did not fully explore the potentialities of the proposed solutions. For example, EIRs can show vaccination rates and integrate with supply data for real-time insights, but the reviewed studies focusing on EIRs solely assessed data quality. Nonetheless, this aspect remains crucial, as 75.5% of the vaccination coverage figures officially reported through the traditional paper system were at least 10% higher than the valid estimates obtained from national surveys.^90^ Additionally, most studies had small samples, limited follow-up, and were region-specific, mainly serving as proof of concept. Except for EIRs, no intervention was scaled nationally, hindering assessment of their impact in complex health systems and questioning long-term sustainability. There is some evidence that shows declining data accuracy over time in EIR in Mongolia.^56^

Furthermore, none of the studies reviewed addressed concerns about data protection, which is likely to be a critical issue in the future, nor did they mention the use of Digital Public Goods—products that meet standards to ensure open and free availability for the public good.^91^ This lack points to a critical gap in research. Moreover, most of the reviewed interventions lacked a cost-effectiveness analysis, which is a key method to inform decision-making regarding the adoption of digital health interventions.

We believe that exploring more context-appropriate data security frameworks, which address core privacy concerns without imposing excessive costs, is a key step toward maximising ICT's potential in limited resource settings. It is essential to include cost-effectiveness analyses in future evaluations and impact studies to prioritise investments and guide policy, particularly in resource-limited systems, where novel approaches must demonstrate their potential for long-term sustainability. In our review, only two studies included a cost-effectiveness analysis.

Our findings highlight the need for further research on effectively integrating advanced ICT solutions into immunisation programmes. The recent introduction of the WHO SMART guidelines^92^ is expected to stimulate more studies but, beyond these guidelines, there is also a need for refined methodologies to evaluate ICT's potential. Traditional protocols like CONSORT may not suffice due to the dynamic and diverse nature of digital health technologies, ranging from telemedicine to AI diagnostics. The implementation of new electronic immunisation registries (eIR) and electronic logistics management information systems (eLMIS) in four LMICs shows the potential to enhance immunisation programme performance at lower costs.^93^ Newest technologies vary in function, implementation, user interaction, and impact on healthcare outcomes, suggesting that the uniform approach of traditional evaluation methods may be inadequate due to the inherent heterogeneity in this field. For instance, Silberman et al. identified 78 potential frameworks to assess digital health technology,^94^ yet none are currently considered standard, although some, like iCHECK-DH^95^ and DEFINED,^94^^,^^95^ show promise. Consistent with the “Principles for Digital Development”,^23^ a novel methodology for assessing digital health should include adaptability, user engagement, data privacy, interoperability, scalability, and sustainability measured through specific indicators. Focusing on the solution rather than the overall health programme allows a more direct evaluation of ICT's effectiveness and clarify the specific contributions of the technology to health outcomes and facilitate benchmarking.

### Caveats and limitations

Our study has several limitations. First, the rapidly changing ICT terminologies and inclusion of only English literature could have impacted our literature search. We therefore broadened our search to include various ICT aspects, covering both old and new developments. We also continuously updated our comprehensive search terms and tried to include all relevant English literature. To ensure the quality of grey literature, we followed the AACODS checklist improving the reliability of our selection process. Second, despite using the PICOS framework, there is a risk of subjective bias in study selection, data extraction, and interpretation. We mitigated this by reaching consensus among authors, with a third author consulted in case of disagreements. Further, the observed asymmetry in the funnel plots suggests a possible publication bias, where positive or significant results are more likely to be published, leading to a higher prevalence of studies with elevated odds ratios on the right side of the plot. Additionally, the presence of heterogeneity among the studies could also account for the observed asymmetry. Thirdly, our meta-analysis concentrated on two outcomes: the DPT-3/Penta-3 coverage rate and the full immunisation rate at one year giving results with high heterogeneity. To address this, we performed subgroup analyses to comprehend the sources of this heterogeneity. Additionally, we would like to point out the lack of clarity in the presentation and interpretation of results from some of the mixed-methods studies as this may hinder the transparency and replicability of the studies. We recommend that future researchers adopt a clearer approach to mixed-methods research by providing a more transparent description on how results obtained through qualitative methods were connected with those gathered through quantitative methods. Based on our quality appraisal, it essential to improve quality of studies on vaccine policies. We encourage a greater focus on methodologically robust research to avoid common pitfalls like unclear data integration, inadequate reporting, and selection biases. Future research should aim to mitigate non-blinding bias by employing designs such as PROBE (Prospective Randomised Open, Blinded Endpoint), which effectively integrates open-label trials with blinded endpoint assessments. This will provide more reliable evidence for policymaking and improve the effectiveness of the vaccination strategies.

### Conclusion

Our study highlights the positive effects of integrating ICT into public health services, specifically immunisation programmes resulting in improvements in terms of record-keeping, supply chain management, and service delivery. Moreover, these technologies can effectively increase the rates of childhood immunisation despite considerable challenges such as the diversity and complexity of ICT interventions. These challenges could be further assessed through a refined systematic framework designed to evaluate the efficiency and adaptability of digital health solutions. Implementing such a framework would be instrumental in fully harnessing the potential of these technologies and enhancing future research.

Our findings also indicate that, in the aftermath of the COVID-19 pandemic, digitalisation of public health systems, specifically that of prevention services such as immunisation programmes—which were most affected by the pandemic given the unprecedented need to a global introduction of a new vaccine—should be central to any initiative of pandemic preparedness and readiness.

## Contributors

Conceptualisation: CEB.

Formal analysis: MZ, HAS, AB, Funding acquisition: CEB.

Methodology: MZ, CEB, AB.

Project administration: HAS.

Supervision: CEB, AB.

Validation: CEB, AB.

Writing–original draft: MZ, AB.

Writing–review & editing: MZ, HAS, RD, HW, TL, CEB, AB.

MZ, HAS and AB have accessed and verified the underlying data in form of the original articles of the included and excluded studies, as well as in form of the data extracted for the meta-analysis. All authors read and approved the final version of the manuscript.

## Data sharing statement

This review is based on publicly available, or fee-based accessible, peer-reviewed manuscripts. Moreover, the authors’ search strategies for PubMed and EMBASE, as well as the search filters for Cochrane library, and BMJ and grey literature have been made available as part of this publication (Supplementary File 2).

## Declaration of interests

The authors declare that there are no conflicts of interest regarding the publication of this manuscript. TL is employed by Deutsche Gesellschaft für Internationale Zusammenarbeit and this article reflects her personal opinion. All other authors have disclosed any financial or personal relationships with individuals or organisations that could potentially influence the work presented in this paper.
