# Supplementary material for: The impact of information and communication technology on immunisation and immunisation programmes in low-income and middle-income countries: a systematic review and meta-analysis
Source: eBioMedicine. 2024 Dec 21;111:105520. doi: 10.1016/j.ebiom.2024.105520 (PMC11732194; doi:10.1016/j.ebiom.2024.105520)
Supplement: Supplementary File 1 [file mmc1.docx]

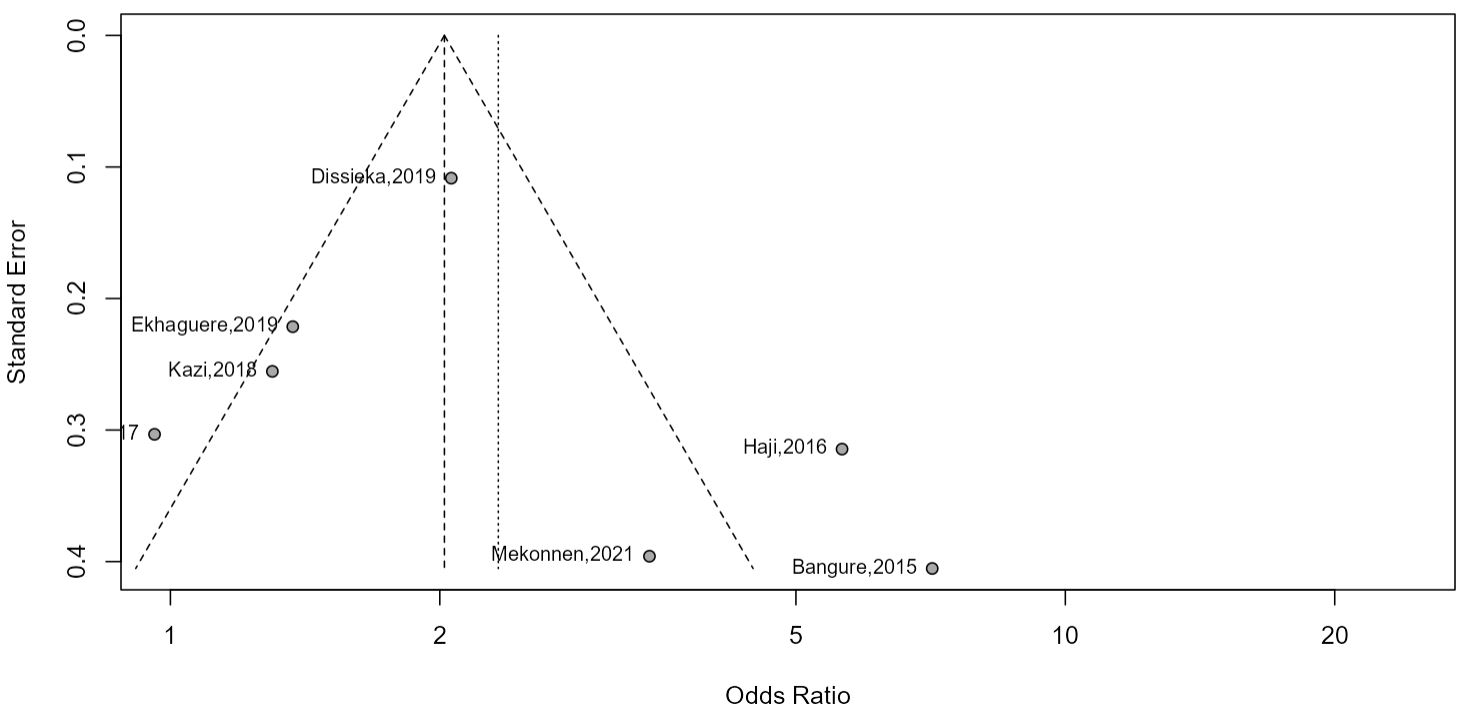


Supplementary file 1a: Funnel plot of analysed studies addressing coverage of Penta 3 vaccine.


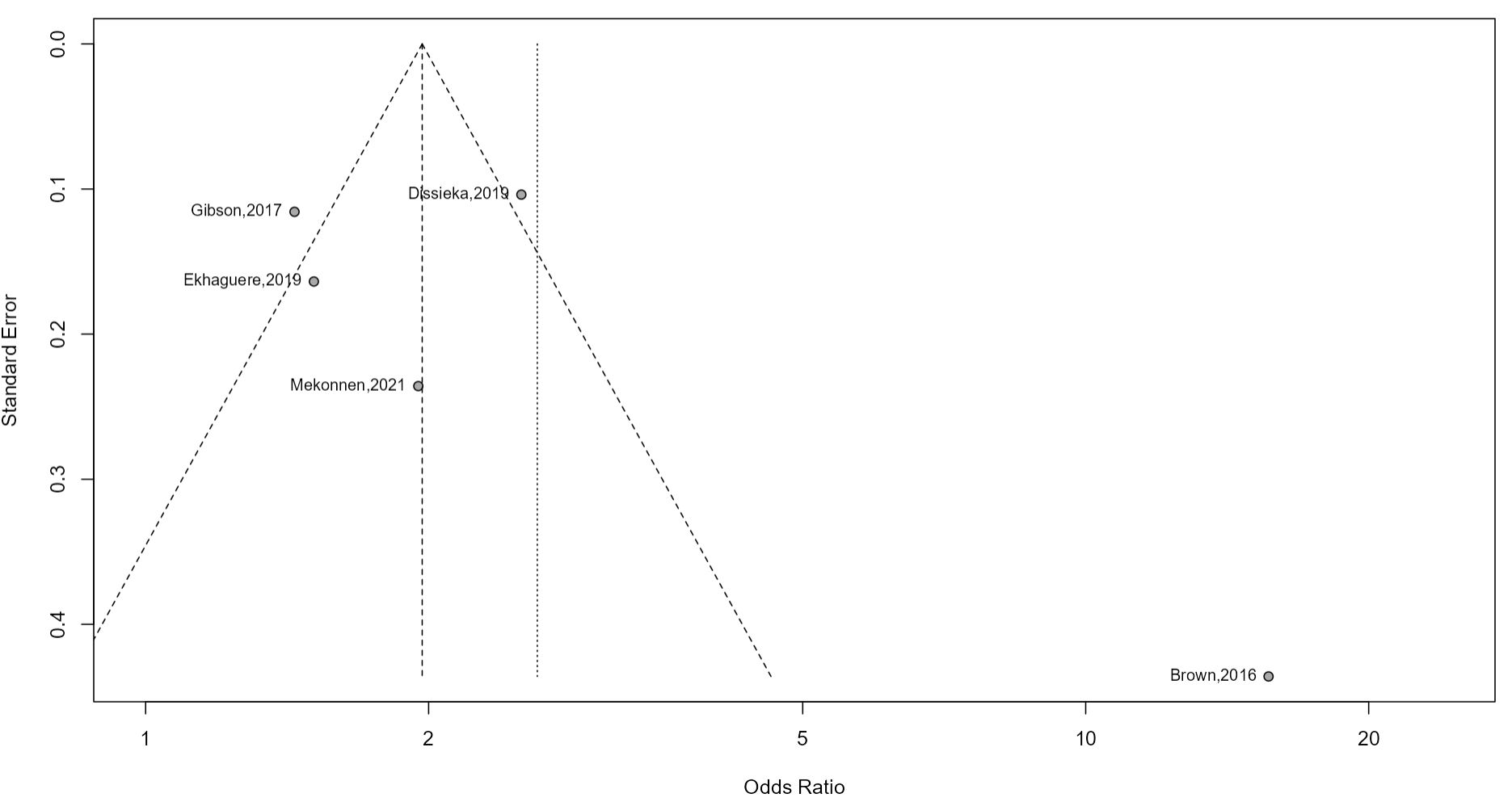


Supplementary file 1b: Funnel plot of analysed studies addressing coverage of full vaccination at one year of age.
