# Supplementary material for: The impact of information and communication technology on immunisation and immunisation programmes in low-income and middle-income countries: a systematic review and meta-analysis
Source: eBioMedicine. 2024 Dec 21;111:105520. doi: 10.1016/j.ebiom.2024.105520 (PMC11732194; doi:10.1016/j.ebiom.2024.105520)
Supplement: Supplementary File 2 [file mmc2.docx]

**Search strategy PubMed**

**PICOS framework**

**Population (P) –** Studies were included that covered immunisation programmes and vaccination campaigns in LMICs as classified by the World Bank (23). Users of these programmes were defined as healthcare workers (HWs), caregivers of the child or health managers.

**Intervention (I) –** Interventions were included that use ICT solutions like electronic health records, Short Message Service (SMS), mHealth, mobile applications, telehealth, telemedicine, software applications, clinical decision support system, health information ex- change, and communication platforms, to enhance the delivery of immunisation services and facilitate the management of immunisation-related information.

**Comparison (C) –** Studies were included that compare ICT interventions with usual care or with interventions that do not use ICT tools.

**Outcome (O) –** Studies were included that evaluate the impact of ICT interventions on immunisation coverage rates, dropout rates, reporting timeliness, data completeness, vaccine stock management, cold chain maintenance, and notification of vaccine side effects.

**Study design (S)** –RCT, quasi-experimental, observational and mixed-method studies were included.

**#A (Lower- and middle-income countries)**

"Developing Countries"[Mesh] OR "low resource setting*"[TIAB] OR "Poverty"[Mesh] OR "Afghanistan"[Mesh] OR "Bangladesh"[Mesh] OR "Benin"[Mesh] OR "Burkina Faso"[Mesh] OR "Burundi"[Mesh] OR "Cambodia"[Mesh] OR "Central African Republic"[Mesh] OR "Chad"[Mesh] OR "Comoros"[Mesh] OR "Democratic Republic of the Congo"[Mesh] OR "Eritrea"[Mesh] OR "Ethiopia"[Mesh] OR "Gambia"[Mesh] OR "Guinea"[Mesh] OR "Guinea-Bissau"[Mesh] OR "Haiti"[Mesh] OR "Kenya"[Mesh] OR "Democratic People's Republic of Korea"[Mesh] OR "Liberia"[Mesh] OR "Madagascar"[Mesh] OR "Malawi"[Mesh] OR "Mali"[Mesh] OR "Mozambique"[Mesh] OR "Myanmar"[Mesh] OR "Nepal"[Mesh] OR "Niger"[Mesh] OR "Rwanda"[Mesh] OR "Sierra Leone"[Mesh] OR "Somalia"[Mesh] OR "Tajikistan"[Mesh] OR "Tanzania"[Mesh] OR "Togo"[Mesh] OR "Uganda"[Mesh] OR "Zimbabwe"[Mesh] OR "Armenia"[Mesh] OR "Bhutan"[Mesh] OR "Bolivia"[Mesh] OR "Cameroon"[Mesh] OR "Cabo Verde"[Mesh] OR "Congo"[Mesh] OR "Cote d'Ivoire"[Mesh] OR "Djibouti"[Mesh] OR "Egypt"[Mesh] OR "El Salvador"[Mesh] OR "Georgia (Republic)"[Mesh] OR "Ghana"[Mesh] OR "Guatemala"[Mesh] OR "Guyana"[Mesh] OR "Honduras"[Mesh] OR "Indonesia"[Mesh] OR "India"[Mesh] OR "Kosovo"[Mesh] OR "Kyrgyzstan"[Mesh] OR "Laos"[Mesh] OR "Lesotho"[Mesh] OR "Mauritania"[Mesh] OR "Micronesia"[Mesh] OR "Moldova"[Mesh] OR "Mongolia"[Mesh] OR "Morocco"[Mesh] OR "Nicaragua"[Mesh] OR "Nigeria"[Mesh] OR "Pakistan"[Mesh] OR "Papua New Guinea"[Mesh] OR "Paraguay"[Mesh] OR "Philippines"[Mesh] OR "Independent State of Samoa"[Mesh] OR "Atlantic Islands"[Mesh] OR "Senegal"[Mesh] OR "Melanesia"[Mesh] OR "Sri Lanka"[Mesh] OR "Sudan"[Mesh] OR "Syria"[Mesh] OR "Timor-Leste"[Mesh] OR "Ukraine"[Mesh] OR "Uzbekistan"[Mesh] OR "Vanuatu"[Mesh] OR "Vietnam"[Mesh] OR "Middle East"[Mesh] OR "Yemen"[Mesh] OR "Zambia"[Mesh] OR "Angola"[Mesh] OR "Albania"[Mesh] OR "Algeria"[Mesh] OR "American Samoa"[Mesh] OR "Argentina"[Mesh] OR "Azerbaijan"[Mesh] OR "Republic of Belarus"[Mesh] OR "Belize"[Mesh] OR "Bosnia and Herzegovina"[Mesh] OR "Botswana"[Mesh] OR "Brazil"[Mesh] OR "Bulgaria"[Mesh] OR "China"[Mesh] OR "Colombia"[Mesh] OR "Costa Rica"[Mesh] OR "Cuba"[Mesh] OR "Dominica"[Mesh] OR "Dominican Republic"[Mesh] OR "Ecuador"[Mesh] OR "Equatorial Guinea"[Mesh] OR "Fiji"[Mesh] OR "Gabon"[Mesh] OR "Grenada"[Mesh] OR "Iran"[Mesh] OR "Iraq"[Mesh] OR "Jamaica"[Mesh] OR "Jordan"[Mesh] OR "Kazakhstan"[Mesh] OR "Lebanon"[Mesh] OR "Libya"[Mesh] OR "Malaysia"[Mesh] OR "Indian Ocean Islands"[Mesh] OR "Mexico"[Mesh] OR "Montenegro"[Mesh] OR "Namibia"[Mesh] OR "Palau"[Mesh] OR "Panama"[Mesh] OR "Peru"[Mesh] OR "Romania"[Mesh] OR "Russia"[Mesh] OR "Serbia"[Mesh] OR "Seychelles"[Mesh] OR "South Africa"[Mesh] OR "Saint Lucia"[Mesh] OR "Saint Vincent and the Grenadines"[Mesh] OR "Suriname"[Mesh] OR "Thailand"[Mesh] OR "Tonga"[Mesh] OR "Tunisia"[Mesh] OR "Turkey"[Mesh] OR "Turkmenistan"[Mesh] OR "Venezuela"[Mesh] OR "Afghanistan"[TIAB] OR "Bangladesh"[TIAB] OR "Benin"[TIAB] OR "Burkina Faso"[TIAB] OR "Burundi"[TIAB] OR "Cambodia"[TIAB] OR "Cabo Verde"[TIAB] OR "Central African Republic"[TIAB] OR "Chad"[TIAB] OR "Comoros"[TIAB] OR "Democratic Republic of the Congo"[TIAB] OR "Eritrea"[TIAB] OR "Ethiopia"[TIAB] OR "Gambia"[TIAB] OR "Guinea"[TIAB] OR "Guinea-Bissau"[TIAB] OR "Haiti"[TIAB] OR "Kenya"[TIAB] OR "Democratic People's Republic of Korea"[TIAB] OR "Liberia"[TIAB] OR "Madagascar"[TIAB] OR "Malawi"[TIAB] OR "Mali"[TIAB] OR "Mozambique"[TIAB] OR "Myanmar"[TIAB] OR "Nepal"[TIAB] OR "Niger"[TIAB] OR "Rwanda"[TIAB] OR "Sierra Leone"[TIAB] OR "Somalia"[TIAB] OR "Tajikistan"[TIAB] OR "Tanzania"[TIAB] OR "Togo"[TIAB] OR "Uganda"[TIAB] OR "Zimbabwe"[TIAB] OR "Armenia"[TIAB] OR "Bhutan"[TIAB] OR "Bolivia"[TIAB] OR "Cameroon"[TIAB] OR "Cape Verde"[TIAB] OR "Congo"[TIAB] OR "Cote d'Ivoire"[TIAB] OR "Djibouti"[TIAB] OR "Egypt"[TIAB] OR "El Salvador"[TIAB] OR "Georgia (Republic)"[TIAB] OR "Ghana"[TIAB] OR "Guatemala"[TIAB] OR "Guyana"[TIAB] OR "Honduras"[TIAB] OR "Indonesia"[TIAB] OR "India"[TIAB] OR "Kiribati"[TIAB] OR "Kosovo"[TIAB] OR "Kyrgyzstan"[TIAB] OR "Kyrgyz"[TIAB] OR "Laos"[TIAB] OR "lao"[TIAB] OR "Lesotho"[TIAB] OR "Mauritania"[TIAB] OR "Micronesia"[TIAB] OR "Moldova"[TIAB] OR "Mongolia"[TIAB] OR "Morocco"[TIAB] OR "Nicaragua"[TIAB] OR "Nigeria"[TIAB] OR "Pakistan"[TIAB] OR "Papua New Guinea"[TIAB] OR "Paraguay"[TIAB] OR "Philippines"[TIAB] OR "Independent State of Samoa"[TIAB] OR "Atlantic Islands"[TIAB] OR "Senegal"[TIAB] OR "Melanesia"[TIAB] OR "Solomon islands"[TIAB] OR "Sri Lanka"[TIAB] OR "Sudan"[TIAB] OR "Swaziland"[TIAB] OR "Syria"[TIAB] OR "East Timor"[TIAB] OR "Timor leste"[TIAB] OR "Ukraine"[TIAB] OR "Uzbekistan"[TIAB] OR "Vanuatu"[TIAB] OR "Vietnam"[TIAB] OR "Middle East"[TIAB] OR "west bank"[TIAB] OR "Gaza"[TIAB] OR "Yemen"[TIAB] OR "Zambia"[TIAB] OR "Angola"[TIAB] OR "Albania"[TIAB] OR "Algeria"[TIAB] OR "Argentina"[TIAB] OR "Samoa"[TIAB] OR "Azerbaijan"[TIAB] OR "Republic of Belarus"[TIAB] OR "Belize"[TIAB] OR "Bosnia-Herzegovina"[TIAB] OR "Botswana"[TIAB] OR "Brazil"[TIAB] OR "Bulgaria"[TIAB] OR "China"[TIAB] OR "Colombia"[TIAB] OR "Costa Rica"[TIAB] OR "Cuba"[TIAB] OR "Dominica"[TIAB] OR "Dominican Republic"[TIAB] OR "Ecuador"[TIAB] OR "Equatorial Guinea"[TIAB] OR "Fiji"[TIAB] OR "Gabon"[TIAB] OR "Grenada"[TIAB] OR "Iran"[TIAB] OR "Iraq"[TIAB] OR "Jamaica"[TIAB] OR "Jordan"[TIAB] OR "Kazakhstan"[TIAB] OR "Lebanon"[TIAB] OR "Libya"[TIAB] OR "Republic of North Macedonia"[TIAB] OR "Malaysia"[TIAB] OR "Indian Ocean Islands"[TIAB] OR "Maldives"[TIAB] OR "Marshall Islands"[TIAB] OR "Mauritius"[TIAB] OR "Mexico"[TIAB] OR "Montenegro"[TIAB] OR "Namibia"[TIAB] OR "Palau"[TIAB] OR "Panama"[TIAB] OR "Peru"[TIAB] OR "Romania"[TIAB] OR "Russia"[TIAB] OR "Russian Federation"[TIAB] OR "Serbia"[TIAB] OR "Seychelles"[TIAB] OR "South Africa"[TIAB] OR "Saint Lucia"[TIAB] OR "Saint Vincent and the Grenadines"[TIAB] OR "Suriname"[TIAB] OR "Thailand"[TIAB] OR "Tonga"[TIAB] OR "Tunisia"[TIAB] OR "Turkey"[TIAB] OR "Turkmenistan"[TIAB] OR "Tuvalu"[TIAB] OR "Venezuela"[TIAB] OR "low resource"[TIAB] OR "under-resourced"[TIAB] OR "resource poor"[TIAB] OR "under-developed"[TIAB] OR "underdeveloped"[TIAB] OR "developing countr*"[TIAB] OR "developing world"[TIAB] OR "third world" [TIAB] OR "lmic*"[TIAB] OR "LMIC*"[Title/Abstract] OR (low[TIAB] AND middle[TIAB] AND income[TIAB]) OR "Africa*"[TIAB] OR "Africa*"[Title/Abstract] OR "Africa"[Mesh]

**#B (Digital Health Technologies)**

"cell phone"[MeSH] OR "cell phone*"[TIAB] OR "cellphone*"[TIAB] OR "Smartphone"[MeSH] OR "Smartphone*"[TIAB] OR "mobile*"[TIAB] OR "telephone"[MeSH]) OR "telephone*"[TIAB] OR "phone"[TIAB] OR "laptop*"[TIAB] OR "computer*"[TIAB] OR "tablet*"[TIAB] OR "text messaging"[MeSH] OR "text messaging"[TIAB] OR "sms"[TIAB] OR "Reminder Systems"[MeSH] OR "reminders*"[TIAB] OR "telemedicine"[MeSH]) OR "telemedicine"[TIAB] OR "mobile applications"[MeSH] OR "software"[MeSH] OR "software"[TIAB] OR "decision support systems, clinical"[MeSH] OR "clinical decision support system*"[TIAB] OR "e-health"[TIAB] OR "ehealth"[TIAB] OR "m-health"[TIAB] OR "mhealth"[TIAB] OR "digital technology"[MeSH] OR "digital*"[TIAB] OR "digitizat*"[TIAB] OR "digitize*"[TIAB] OR "digitizing"[TIAB] OR "Electronic Health Records"[MeSH] OR "medical records systems, computerized"[MeSH] OR "Electronic Health Record*"[TIAB] OR "machine learning"[MeSH] OR "Machine Learning"[TIAB] OR "artificial intelligence"[MeSH] OR "artificial intelligence"[TIAB] OR (medical[TIAB] AND health[TIAB] AND record*[TIAB]) OR "Geographic Information Systems"[MeSH] OR "GIS"[TIAB] OR "DHIS"[TIAB] OR "Registries"[MeSH] OR "registr*"[TIAB] OR “Computers, Handheld” [MesH] OR “Algorithms*” [MesH] OR “electronic”[tiab] OR “algorithm*”[TIAB] OR “application*”[TIAB] OR (“clinical decision”[TIAB] AND (tool*[TIAB] OR tree*[TIAB])) OR "Database Management Systems"[MeSH] OR “app” [TIAB] OR “apps” [TIAB] OR “Internet"[MeSH] OR “Computers, Handheld"[MeSH]OR “Medical Informatics Applications"[MeSH] OR “Therapy, Computer-Assisted"[MeSH] OR “online” [TIAB] OR “web” [TIAB] OR “internet” [TIAB] OR “smartwatch*”[TIAB] OR “device*”[TIAB] OR “technolog*”[TIAB]

**#C (Immunization programs)**

"Immunization*"[TIAB] OR "Immunization Programs"[MeSH] OR “immunisation*"[TIAB] OR "immunization*"[TIAB] OR "Expanded program Immunization"[TIAB] OR "EPI"[TIAB] OR "vaccin*"[TIAB] OR “Mass Vaccination"[MeSH] OR “inoculat*” [TIAB] OR “boost*” [TIAB]

**Search #A AND #B AND #C**

**EMBASE search strategy**

**#A Digital Health:**

'digital health'/exp OR 'digital health technology'/exp OR 'digital health intervention'/exp OR 'telehealth'/exp OR 'telemedicine'/exp OR 'telecommunication'/exp OR 'mobile health'/exp OR 'mobile health application'/exp OR 'mobile application'/exp OR 'medical informatics'/exp OR 'smartphone'/exp OR 'mobile device'/exp OR 'mobile phone'/exp OR 'text message'/exp OR 'reminder system'/exp OR 'telephone'/exp OR 'e-mail'/exp OR 'personal digital assistant'/exp OR 'electronic health record'/exp OR 'digital health':ab,ti OR 'digital health technology':ab,ti OR 'digital health intervention':ti,ab OR 'telehealth':ti,ab OR 'telemedicine':ti,ab OR 'telecommunication':ti,ab OR 'mobile health':ab,ti OR 'mobile health application':ab,ti OR 'mobile application':ti,ab OR 'medical informatics':ti,ab OR 'smartphone':ti,ab OR 'mobile device':ti,ab OR 'mobile phone':ti,ab OR 'text message':ti,ab OR 'reminder system':ti,ab OR 'telephone':ti,ab OR 'e-mail':ti,ab OR 'personal digital assistant':ti,ab OR 'electronic health record':ti,ab

**#B Immunization:**

'immunization'/exp OR 'immunization' OR 'expanded program on immunization'/exp OR 'epi' OR 'preventive health service'/exp OR 'preventive health service' OR 'mass immunization'/exp OR 'mass immunization' OR 'vaccination coverage'/exp OR 'vaccination coverage' OR 'vaccination'/exp OR 'vaccination' OR 'immunization':ab,ti OR 'expanded program on immunization':ab,ti OR 'preventive health service':ti,ab OR 'mass immunization':ti,ab OR 'vaccination coverage':ab,ti OR 'vaccination':ab,ti OR epi:ab,ti

**#C LMIC :**

'low and middle income country'/exp OR 'low and middle income country' OR 'low and middle income countries'/exp OR 'low and middle income countries' OR 'low income country'/exp OR 'low income country' OR 'middle income country'/exp OR 'middle income country' OR 'low resource setting'/exp OR 'low resource setting' OR 'developing country'/exp OR 'developing country' OR 'afghanistan'/exp OR 'afghanistan' OR 'bangladesh'/exp OR 'bangladesh' OR 'benin'/exp OR 'benin' OR 'burkina faso'/exp OR 'burkina faso' OR 'burundi'/exp OR 'burundi' OR 'cambodia'/exp OR 'cambodia' OR 'central african republic'/exp OR 'central african republic' OR 'chad'/exp OR 'chad' OR 'comoros'/exp OR 'comoros' OR 'democratic republic congo'/exp OR 'democratic republic congo' OR 'congo'/exp OR 'congo' OR 'eritrea'/exp OR 'eritrea' OR 'ethiopia'/exp OR 'ethiopia' OR 'gambia'/exp OR 'gambia' OR 'guinea'/exp OR 'guinea' OR 'guinea-bissau'/exp OR 'guinea-bissau' OR 'haiti'/exp OR 'haiti' OR 'kenya'/exp OR 'kenya' OR 'north korea'/exp OR 'north korea' OR 'liberia'/exp OR 'liberia' OR 'madagascar'/exp OR 'madagascar' OR 'malawi'/exp OR 'malawi' OR 'mozambique'/exp OR 'mozambique' OR 'myanmar'/exp OR 'myanmar' OR 'nepal'/exp OR 'nepal' OR 'niger'/exp OR 'niger' OR 'nigeria'/exp OR 'nigeria' OR 'rwanda'/exp OR 'rwanda' OR 'sierra leone'/exp OR 'sierra leone' OR 'somalia'/exp OR 'somalia' OR 'tajikistan'/exp OR 'tajikistan' OR 'tanzania'/exp OR 'tanzania' OR 'togo'/exp OR 'togo' OR 'uganda'/exp OR 'uganda' OR 'zimbabwe'/exp OR 'zimbabwe' OR 'armenia'/exp OR 'armenia' OR 'bhutan'/exp OR 'bhutan' OR 'bolivia'/exp OR 'bolivia' OR 'cameroon'/exp OR 'cameroon' OR 'cape verde'/exp OR 'cape verde' OR 'cote d`ivoire'/exp OR 'cote d`ivoire' OR 'djibouti'/exp OR 'djibouti' OR 'egypt'/exp OR 'egypt' OR 'el salvador'/exp OR 'el salvador' OR 'georgia (republic)'/exp OR 'georgia (republic)' OR 'ghana'/exp OR 'ghana' OR 'guatemala'/exp OR 'guatemala' OR 'guyana'/exp OR 'guyana' OR 'honduras'/exp OR 'honduras' OR 'indonesia'/exp OR 'indonesia' OR 'india'/exp OR 'india' OR 'kosovo'/exp OR 'kosovo' OR 'kyrgyzstan'/exp OR 'kyrgyzstan' OR 'laos'/exp OR 'laos' OR 'lesotho'/exp OR 'lesotho' OR 'mauritania'/exp OR 'mauritania' OR 'federated states of micronesia'/exp OR 'federated states of micronesia' OR 'moldova'/exp OR 'moldova' OR 'mongolia'/exp OR 'mongolia' OR 'nicaragua'/exp OR 'nicaragua' OR 'pakistan'/exp OR 'pakistan' OR 'papua new guinea'/exp OR 'papua new guinea' OR 'philippines'/exp OR 'philippines' OR 'samoa'/exp OR 'samoa' OR 'sao tome and principe'/exp OR 'sao tome and principe' OR 'senegal'/exp OR 'senegal' OR 'solomon islands'/exp OR 'solomon islands' OR 'sri lanka'/exp OR 'sri lanka' OR 'sudan'/exp OR 'sudan' OR 'swaziland'/exp OR 'swaziland' OR 'syrian arab republic'/exp OR 'syrian arab republic' OR 'timor-leste'/exp OR 'timor-leste' OR 'ukraine'/exp OR 'ukraine' OR 'uzbekistan'/exp OR 'uzbekistan' OR 'vanuatu'/exp OR 'vanuatu' OR 'viet nam'/exp OR 'viet nam' OR 'yemen'/exp OR 'yemen' OR 'zambia'/exp OR 'zambia' OR 'angola'/exp OR 'angola' OR 'albania'/exp OR 'albania' OR 'algeria'/exp OR 'algeria' OR 'american samoa'/exp OR 'american samoa' OR 'argentina'/exp OR 'argentina' OR 'azerbaijan'/exp OR 'azerbaijan' OR 'belarus'/exp OR 'belarus' OR 'belize'/exp OR 'belize' OR 'bosnia and herzegovina'/exp OR 'bosnia and herzegovina' OR 'botswana'/exp OR 'botswana' OR 'brazil'/exp OR 'brazil' OR 'bulgaria'/exp OR 'bulgaria' OR 'costa rica'/exp OR 'costa rica' OR 'cuba'/exp OR 'cuba' OR 'dominica'/exp OR 'dominica' OR 'dominican republic'/exp OR 'dominican republic' OR 'ecuador'/exp OR 'ecuador' OR 'equatorial guinea'/exp OR 'equatorial guinea' OR 'fiji'/exp OR 'fiji' OR 'gabon'/exp OR 'gabon' OR 'grenada'/exp OR 'grenada' OR 'iran'/exp OR 'iran' OR 'iraq'/exp OR 'iraq' OR 'jamaica'/exp OR 'jamaica' OR 'jordan'/exp OR 'jordan' OR 'kazakhstan'/exp OR 'kazakhstan' OR 'lebanon'/exp OR 'lebanon' OR 'libyan arab jamahiriya'/exp OR 'libyan arab jamahiriya' OR 'macedonia (republic)'/exp OR 'macedonia (republic)' OR 'malaysia'/exp OR 'malaysia' OR 'maldives'/exp OR 'maldives' OR 'mexico'/exp OR 'mexico' OR 'montenegro (republic)'/exp OR 'montenegro (republic)' OR 'namibia'/exp OR 'namibia' OR 'palau'/exp OR 'palau' OR 'panama'/exp OR 'panama' OR 'peru'/exp OR 'peru' OR 'romania'/exp OR 'romania' OR 'russian federation'/exp OR 'russian federation' OR 'serbia'/exp OR 'serbia' OR 'seychelles'/exp OR 'seychelles' OR 'south africa'/exp OR 'south africa' OR 'saint lucia'/exp OR 'saint lucia' OR 'saint vincent and the grenadines'/exp OR 'saint vincent and the grenadines' OR 'suriname'/exp OR 'suriname' OR 'thailand'/exp OR 'thailand' OR 'tonga'/exp OR 'tonga' OR 'tunisia'/exp OR 'tunisia' OR 'turkey (republic)'/exp OR 'turkey (republic)' OR 'turkmenistan'/exp OR 'turkmenistan' OR 'venezuela'/exp OR 'venezuela' OR 'afghanistan':ab,ti OR 'bangladesh':ab,ti OR 'benin':ab,ti OR 'burkina faso':ab,ti OR 'burundi':ab,ti OR 'cambodia':ab,ti OR 'cabo verde':ab,ti OR 'central african republic':ab,ti OR 'chad':ab,ti OR 'comoros':ab,ti OR 'eritrea':ab,ti OR 'ethiopia':ab,ti OR 'gambia':ab,ti OR 'guinea':ab,ti OR 'haiti':ab,ti OR 'kenya':ab,ti OR 'korea':ab,ti OR 'liberia':ab,ti OR 'madagascar':ab,ti OR 'malawi':ab,ti OR 'mali':ab,ti OR 'mozambique':ab,ti OR 'myanmar':ab,ti OR 'nepal':ab,ti OR 'niger':ab,ti OR 'rwanda':ab,ti OR 'sierra leone':ab,ti OR 'somalia':ab,ti,ca OR 'tajikistan':ab,ti,ca OR 'tanzania':ab,ti OR 'togo':ab,ti OR 'uganda':ab,ti OR 'zimbabwe':ab,ti OR 'armenia':ab,ti OR 'bhutan':ab,ti OR 'bolivia':ab,ti OR 'cameroon':ab,ti OR 'cape verde':ab,ti OR 'congo':ab,ti OR 'cote divoire':ab,ti OR 'ivory coast':ab,ti OR 'djibouti':ab,ti OR 'egypt':ab,ti OR 'el salvador':ab,ti OR 'georgia':ab,ti OR 'ghana':ab,ti OR 'guatemala':ab,ti OR 'guyana':ab,ti OR 'honduras':ab,ti OR 'indonesia':ab,ti OR 'india':ab,ti OR 'kiribati':ab,ti OR 'kosovo':ab,ti OR 'kyrgyzstan':ab,ti OR 'kyrgyz':ab,ti OR 'lao':ab,ti OR 'lesotho':ab,ti OR 'mauritania':ab,ti OR 'micronesia':ab,ti OR 'moldova':ab,ti OR 'mongolia':ab,ti OR 'morocco':ab,ti OR 'nicaragua':ab,ti OR 'nigeria':ab,ti OR 'pakistan':ab,ti OR 'papua new guinea':ab,ti OR 'paraguay':ab,ti OR 'philippines':ab,ti OR 'atlantic islands':ab,ti OR 'sao tome':ab,ti OR principe:ab,ti OR 'senegal':ab,ti OR 'melanesia':ab,ti OR 'solomon islands':ab,ti OR 'sri lanka':ab,ti OR 'sudan':ab,ti OR 'swaziland':ab,ti OR 'syria':ab,ti OR 'east timor':ab,ti OR 'timor leste':ab,ti OR 'ukraine':ab,ti OR 'uzbekistan':ab,ti OR 'vanuatu':ab,ti OR 'vietnam':ab,ti OR 'middle east':ab,ti OR 'west bank':ab,ti OR 'gaza':ab,ti OR 'yemen':ab,ti OR 'zambia':ab,ti OR 'angola':ab,ti OR 'albania':ab,ti OR 'algeria':ab,ti OR 'argentina':ab,ti OR 'samoa':ab,ti OR 'azerbaijan':ab,ti OR 'republic of belarus':ab,ti OR 'belize':ab,ti OR bosnia:ab,ti OR herzegovina:ab,ti OR 'botswana':ab,ti OR 'brazil':ab,ti OR 'bulgaria':ab,ti OR 'costa rica':ab,ti OR 'cuba':ab,ti OR 'dominica':ab,ti OR 'dominican republic':ab,ti OR 'ecuador':ab,ti OR 'equatorial guinea':ab,ti OR 'fiji':ab,ti OR 'gabon':ab,ti OR 'grenada':ab,ti OR 'iran':ab,ti OR 'iraq':ab,ti OR 'jamaica':ab,ti OR 'jordan':ab,ti OR 'kazakhstan':ab,ti OR 'lebanon':ab,ti OR 'libya':ab,ti OR 'macedonia':ab,ti OR 'malaysia':ab,ti OR 'indian ocean islands':ab,ti OR 'maldives':ab,ti OR 'marshall islands':ab,ti OR 'mauritius':ab,ti OR 'mexico':ab,ti OR 'montenegro':ab,ti OR 'namibia':ab,ti OR 'palau':ab,ti OR 'panama':ab,ti OR 'peru':ab,ti OR 'romania':ab,ti OR 'russia':ab,ti OR 'russian federation':ab,ti OR 'serbia':ab,ti OR 'seychelles':ab,ti OR 'south africa':ab,ti OR 'saint lucia':ab,ti OR 'saint vincent and the grenadines':ab,ti OR 'suriname':ab,ti OR 'thailand':ab,ti OR 'tonga':ab,ti OR 'tunisia':ab,ti OR 'turkey':ab,ti OR 'turkmenistan':ab,ti OR 'tuvalu':ab,ti OR 'venezuela':ab,ti OR 'low resource':ab,ti OR 'under resourced':ab,ti OR 'resource poor':ab,ti OR 'under developed':ab,ti OR 'underdeveloped*':ab,ti OR 'developing country':ab,ti OR 'developing* countries':ab,ti OR 'developing world':ab,ti OR 'third world':ab,ti OR lmic*:ab,ti OR (low:ab,ti AND middle:ab,ti AND income:ab,ti)

Filters: 2010 to 2023, Language- English

Search: #A AND #B AND #C

**Search Filters for Cochrane library**

**A Digital health:**

1 Mobile Applications/

2 exp Internet/

3 exp Cell Phone/

4 exp Computers, Handheld/

5 Medical Informatics Applications/

6 Therapy, Computer-Assisted/

7 (app or apps).ti,ab.

8 (online or web or internet or digital*).ti.

9 ((online or web or internet or digital*) adj3 (based or application* or intervention* or program* or therap*)).ab.

10 (phone* or telephone* or smartphone* or cellphone* or smartwatch*).ti.

11 ((phone* or telephone* or smartphone* or cellphone* or smartwatch*) adj3 (based or application* or intervention* or program* or therap*)).ab.

12 (mobile health or mhealth or m-health or ehealth or e-health or emental or e-mental).ti.

13 ((mobile health or mhealth or m-health or ehealth or e-health or emental or e-mental) adj3 (based or application* or intervention* or program* or therap*)).ab.

14 (mobile* adj3 (based or application* or intervention* or device* or technolog*)).ti,ab.

15 or/1-14

AND

**B Immunization**

Vaccination* or Immunization* or EPI or vaccination* or programs/ Immunization* programs or Expanded program in immunization*or vaccination* coverage or Immunization* coverage

AND

**C Low- and middle-income countries**

Low- and middle-income countries* or LMIC* or Developing Countries or resource-limited settings or low-income countries or middle-income countries or upper middle-income countries

**Search: #A AND #B AND #C**

**Search filters for BMJ and GREY Literature**

**Keywords**

**Digital Health Keywords:**

eHealth or mHealth or health technology or health informatics or telemedicine

**Immunization keywords:**

Vaccination or immunization programs or vaccine coverage or vaccine delivery systems or immunization rates

**Low- and middle-income countries keywords:**

developing countries or resource-limited settings or LMICs or Low- and middle-income countries
