# Supplementary material for: The impact of information and communication technology on immunisation and immunisation programmes in low-income and middle-income countries: a systematic review and meta-analysis
Source: eBioMedicine. 2024 Dec 21;111:105520. doi: 10.1016/j.ebiom.2024.105520 (PMC11732194; doi:10.1016/j.ebiom.2024.105520)
Supplement: Supplementary File 3 [file mmc3.docx]

Supplementary file 3: List of excluded studies from the systematic review

| **Authors** | **Title** | **Year** | **Country** | **Study Design** | **Exclusion reason** |
| --- | --- | --- | --- | --- | --- |
| Clarke et al (2019) | Strengths, pitfalls, and lessons learned in implementing electronic collection of childhood vaccination data in Zambia: The SmartCare experience. | 2019 | Zambia | Cross sectional | Intervention implementation failure (2%). |
| Schlumberger et al (2015) | Positive impact on the expanded program on immunisation when sending call-back SMS through a computerized immunisation register, Bobo Dioulasso (Burkina Faso) | 2015 | Burkina Faso | RCT | Excessive loss to follow-up^[[1]](#footnote-1)^ |
| Kazi (2014) | Monitoring polio supplementary immunization activities using an automated short text messaging system in Karachi, Pakistan. | 2014 | Pakistan | cRCT | Excessive loss to follow-up |
| Lee et al (2022) | Re-designing the Mozambique vaccine supply chain to improve access to vaccines. | 2022 | Mozambique | Experimental | Duplication |
| Brown (2017) | Feasibility of implementing a cellphone-based reminder/recall strategy to improve childhood routine immunization in a low-resource setting: a descriptive report | 2017 | Nigeria | RCT | Duplication |
| Busso (2015) | Did you get your shots? Experimental evidence on the role of reminders | 2015 | Guatemala | RCT | No digital intervention |
| Mokaya et al (2017) | Use of cellular phone contacts to increase return rates for immunization services in Kenya | 2017 | Kenya | Quasi experimental | No digital intervention |
| Lamanna et al (2019) | A pilot study of a novel, incentivised mHealth technology to monitor the vaccine supply chain in rural Zambia | 2018 | Zambia | Experimental | No digital intervention |
| Levine et al (2021) | Mobile nudges and financial incentives to improve coverage of timely neonatal vaccination in rural areas (GEVaP trial): A 3-armed cluster randomized controlled trial in Northern Ghana | 2021 | Ghana | cRCT | No digital intervention |
| Ateudjieu et al (2022) | Tracking Demographic Movements and Immunization Status to Improve Children's Access to Immunization: Field-Based Randomized Controlled Trial | 2022 | Cameroon | RCT | No digital intervention |
| Ghadieh et al (2015) | The effect of various types of patients’ reminders on the uptake of pneumococcal vaccine in adults: A randomized controlled trial | 2015 | Lebanon | RCT | No immunisation program |
| Murthy et al (2019) | The Impact of an mHealth Voice Message Service (mMitra) on Infant Care Knowledge, and Practices Among Low-Income Women in India: Findings from a Pseudo-Randomized Controlled Trial | 2019 | India | RCT | No immunisation program |
| Wakadha et al (2013) | The feasibility of using mobile-phone based SMS reminders and conditional cash transfers to improve timely immunization in rural Kenya | 2013 | Kenya | Protocol | No or wrong comparator |
| Baron (2013) | Use of a text message-based pharmacovigilance tool in Cambodia: pilot study. | 2013 | Cambodia | Pilot study | No or wrong comparator |
| Teng et al (2014) | Using Mobile Health (mHealth) and geospatial mapping technology in a mass campaign for reactive oral cholera vaccination in rural Haiti. | 2014 | Haiti | Experimental | No or wrong comparator |
| Kaewkungwal et al (2015) | Application of mobile technology for improving expanded program on immunization among highland minority and stateless populations in northern Thailand border. | 2015 | Thailand | Quasi-experimental | No or wrong comparator |
| Haskew et al (2015) | Use of Mobile Information Technology during Planning, Implementation and Evaluation of a Polio Campaign in South Sudan. | 2015 | Sudan | Observational | No or wrong comparator |
| Touray et al (2016) | Tracking Vaccination Teams During Polio Campaigns in Northern Nigeria by Use of Geographic Information System Technology: 2013-2015. | 2016 | Nigeria | Experimental | No or wrong comparator |
| Uddin et al (2015) | Use of mobile phones for improving vaccination coverage among children living in rural hard-to-reach areas and urban streets of Bangladesh. | 2015 | Bangladesh | Quasi experimental | No or wrong comparator |
| Chen et al (2016) | Effectiveness of a smartphone app on improving immunization of children in rural Sichuan Province, China: a cluster randomized controlled trial | 2016 | China | cRCT | No or wrong comparator |
| Oh et al (2016) | Real-Time Monitoring of Vaccination Campaign Performance Using Mobile Phones - Nepal, 2016. | 2016 | Nepal | Experimental | No or wrong comparator |
| Gilbert (2017) | Assessing stability and performance of a digitally enabled supply chain: Retrospective of a pilot in Uttar Pradesh, India. | 2017 | India | Retrospective cohort pilot | No or wrong comparator |
| Chandir (2017) | Feasibility of using global system for mobile communication (GSM)-based tracking for vaccinators to improve oral poliomyelitis vaccine campaign coverage in rural Pakistan | 2017 | Pakistan | Pilot study | No or wrong comparator |
| Nagar et al (2017) | A cluster randomized trial to determine the effectiveness of a novel, digital pendant and voice reminder platform on increasing infant immunization adherence in rural Udaipur, India | 2017 | India | cRCT | No or wrong comparator |
| Afzal et al (2017) | An effective and doable interventional strategy to enhance vaccination coverage - are we ready to change? | 2017 | Pakistan | Prospective, interventional study | No or wrong comparator |
| Sayuri Sato et al (2018) | Use of electronic immunization registry in the surveillance of adverse events following immunization. | 2018 | Brazil | Descriptive study | No or wrong comparator |
| Jusril et al (2020) | Digital health for real-time monitoring of a national immunization campaign in Indonesia: a large-scale effectiveness evaluation | 2020 | Indonesia | Observational | No or wrong comparator |
| Kenna et al (2021) | Leapfrogging with technology: introduction of a monitoring platform to support a large-scale Ebola vaccination program in Rwanda. | 2021 | Rwanda | Experimental | No or wrong comparator |
| Chakraborty et al (2021) | Does exposure to health information through mobile phones increase immunization knowledge, completeness and timeliness in rural India? | 2021 | India | RCT | No or wrong comparator |
| Yunusa et al (2022) | Effect of mobile phone text message and call reminders in the completeness of pentavalent vaccines in Kano state, Nigeria. | 2022 | Nigeria | Quasi experimental | No or wrong comparator |
| Mohammed Bello et al (2021) | Real-time monitoring of a circulating vaccine-derived poliovirus outbreak immunization campaign using digital health technologies in South Sudan. | 2021 | Sudan | cluster randomised trial ? | No or wrong comparator |
| Siddique et al (2023) | Using geographic information system to track children and optimize immunization coverage and equity in Karachi, Pakistan | 2023 | Pakistan | Observational | No or wrong comparator |
| Gibson et al (2016) | The Mobile Solutions for Immunization (M-SIMU) Trial: A Protocol for a Cluster Randomized Controlled Trial That Assesses the Impact of Mobile Phone Delivered Reminders and Travel Subsidies to Improve Childhood Immunization Coverage Rates and Timeliness in | 2016 | Kenya | RCT | Only protocol published |
| Mekonnen et al (2019) | Effect of mobile phone text message reminders on improving completeness and timeliness of routine childhood vaccinations in North-West, Ethiopia: a study protocol for randomised controlled trial | 2019 | Ethiopia | RCT | Only protocol published |
| Kazi et al (2017) | Geo-spatial reporting for monitoring of household immunization coverage through mobile phones: Findings from a feasibility study. | 2017 | Pakistan | cRCT | Wrong study esign |

^[[2]](#footnote-2)^

1. For Major bias: one study presented an implementation failure outside the used ICT. We were therefore not able to evaluate its impact.

   Two studies reported major loss to follow-up. Unsuitable study design: one study reported only the study protocol and another one only the development of the intervention [↑](#footnote-ref-1)
2. For Major bias: one study presented an implementation failure outside the used ICT. We were therefore not able to evaluate its impact. Two studies reported major loss to follow-up. Unsuitable study design: one study reported only the study protocol and another one only the development of the intervention [↑](#footnote-ref-2)
