# Supplementary material for: The impact of information and communication technology on immunisation and immunisation programmes in low-income and middle-income countries: a systematic review and meta-analysis
Source: eBioMedicine. 2024 Dec 21;111:105520. doi: 10.1016/j.ebiom.2024.105520 (PMC11732194; doi:10.1016/j.ebiom.2024.105520)
Supplement: Supplementary File 4 [file mmc4.docx]

Supplementary file 4: Summary of included studies

| **Study** | **Population and Sample Size** | **Country** | **Study Design** | **Intervention** | **Main Outcome** | **Conclusion** |
| --- | --- | --- | --- | --- | --- | --- |
| Kawakatsu et al (2020)^30^ | n=12,175 children | Nigeria | Randomised controlled trial | SMS Text reminders | Return rate for child vaccinations | The intervention group's return rate was consistently 4.8% - 6.0% higher than the control group's (p < 0.001) across all five timings (on schedule, and 7, 14, 30 days, and 3 months post-appointment) |
| Bangure et al (2015)^31^ | n=304 children | Zimbabwe | Randomised controlled trial | SMS Text reminders | Full coverage rate at 6, 10 and 14 weeks | Immunisation coverage at 6, 10, and 14 weeks was 97%, 96%, and 95% in the intervention group, and 82%, 80%, and 75% in the non-intervention group, respectively (p < 0.001 for all comparisons). Those who did not delay receiving immunisation at 14 weeks was 82% for the intervention and 8% for non-intervention group |
| Kagucia et al (2021)^32^ | n=537 children | Kenya | Randomised controlled trial | SMS text reminders + monetary incentive | MCV-1 on-time vaccination rate | Timely MCV1 coverage was 68% in the control arm, 78% in each intervention arm. The increase in the SMS arm was not statistically significant (adjusted relative risk 1.13; 95% CI 0.99-1.30), but it was significant in the SMS + 150 KES arm (1.16; 95% CI 1.01-1.32) |
| Tsafack et al (2015)^33^ | n=471 children | Cameroon | Randomised controlled trial | beep (short phone call not picked up) | AEFIs reporting rate | The AEFIs reporting rate in the intervention group was significantly higher than that in the control group [RR = 18.9; CI95 (2.5; 140.0) (P=0.0004) |
| Seth et al (2018)^34^ | n=549 children | India | Randomised controlled trial | SMS text reminders (through cloud based biometric software platform) | Full coverage coverage rate at 24 months | At enrollment, immunisation coverage was 33% across all groups. By the study's end, it increased to 41.7% (IQR: 23.1%-69.2%) in the control group, 40.1% (IQR: 30.8%-69.2%) in the mobile phone reminder group, and 50.0% (IQR: 30.8%-76.9%) in the compliance-linked incentives group |
| Gibson et al (2017)^35^ | n=152 children | Kenya | Cluster Randomised controlled trial | SMS text reminders + monetary incentive | Full coverage rate at 12 months of age | Full immunisation was 82% in the control group, 86% in both the SMS and SMS + 75KES groups, and 90% in the SMS + 200KES group. Children in the SMS + 200KES group had a higher likelihood of full immunisation (relative risk 1.09, 95% CI 1.02-1.16) compared to the control group |
| Dissieka et al (2019)^36^ | n=1,596 children | Côte d'Ivoire | Randomised controlled trial | voice or SMS text reminders | Penta-3 coverage rate MMR/yellow fever coverage rate | Infants in the intervention group were 2.85 (95% CI: 1.85-4.37), 2.80 (95% CI: 1.88-4.17), 2.68 (95% CI: 1.84-3.91), and 4.52 (95% CI: 2.84-7.20) times more likely to receive pentavalent 1-3 and MMR/yellow fever doses, respectively. |
| Haji et al (2016)^37^ | n=1,116 children | Kenya | Randomised controlled trial | SMS text reminders | Dropout rate | Less drop out in the intervention group compared to control group (OR 0.2, 95% CI: 0.04-0.8) |
| Ateudjieu et al (2014)^38^ | n=348 children | Cameroon | Randomised controlled trial | SMS text reminders | AEFIs reporting rate | Not significant reporting rate increase in intervention group (RR=1.4; 95% CI:0.8-1.6) |
| Ekhaguere et al (2019)^39^ | n=600 children | Nigeria | Randomised controlled trial | SMS text reminders | Full coverage rate at 18 weeks of age | Intervention group was more likely to receive Penta-3 (84% vs 78%, RR 1.09, 95% CI 1.01 to 1.17), measles (73% vs 65%, RR 1.13, 95% CI 1.02 to 1.26) and all scheduled immunisations collectively (57% vs 47%, RR 1.13, 95% CI 1.02 to 1.26) within 1 week of the recommended date |
| Mekonnen et al (2021)^40^ | n=434 children | Ethiopia | Randomised controlled trial | SMS Text reminders | Full coverage rate at 12 months of age On-time vaccination rate | Intervention group had higher rates of Penta-3 (95.8% vs 86.9%; P<.001), measles (91.5% vs 79.3%; P<.001), and full vaccination (82.6% vs 70.9%; P=.002). More children in the intervention group received Penta-3 (88.7% vs 69.2%; P<.001), measles (87.1% vs 68.6%; P<.001), and all scheduled vaccinations on time (63.3% vs 39.9%; P<.001) |
| Brown et al (2016)^41^ | n= 595 children | Nigeria | Randomised controlled trial | Immunisation reminder calls + specific training | Full coverage rate at 12 months of age | Full coverage rate was 98.6%, 97.3% and 57.3% for phone calls, phone calls + training and control, respectively. Compared to the control group, the cellphone R/R group was 72 % (RR 1.72, 95 % CI 1.50-1.98) and the RR+ training group 70 % (RR 1.70, 95 % CI 1.47-1.95) more likely to complete immunisation |
| Eze at al (2015)^42^ | n=905 children | Nigeria | Randomised controlled trial | SMS Text reminders | DPT-3 coverage rate at 18 weeks of age | Children in the intervention group were 1.5 times earlier in their receipt of DPT-3. Immunisation coverage was also 8.7% better in the intervention group |
| Domek et al (2019)^43^ | n=720 children | Guatemala | Randomised controlled trial | SMS Text reminders | Timeliness of the second and third visits of the primary immunisation series. | Intervention participants presented on the scheduled date more often for visit 2 (42.2% vs. 30.7%, p = <.001) and for visit 3 (34.0% vs. 27.0%, p = 0.05) |
| Kazi et al (2018)^44^ | n=300 children | Pakistan | Randomised controlled trial | SMS Text reminders | Full coverage rate at 18 weeks of age | The immunisation coverage was consistently higher in the intervention group (ITT analyses) at the 6 weeks scheduled visit (76.0% vs 71.3%, P=.36). The 10 weeks scheduled visit (58.7% vs 52.7%, P=.30) and the 14 weeks scheduled visit (31.3% vs 26.0%, P=0.31) |
| Prosser et al (2017)^45^ | 237 households in Mozambique  37 health facilities in Benin | Benin and Mozambique | qualitative assessment | HERMES (Simulation modeling Tool) | Benin: EVM performance score Mozambique: DTP-3 coverage rate, DTP dropout rate and stock outs | Benin: On the base of the EVM performance score the distribution score increased from 40% to 100% (control 32%); vaccine management practices increased from 58% to 94%; and infrastructure increased from 55% to 94% (control 63%) with the procurement of improved cold chain equipment. Mozambique: DTP-3 coverage rate increased from 68.9% to 92.8% (OR 5.8, 95% CI 3.2–10.5). Drop-out rates between DTP1 and DTP3 decreased from 12% to 3.8%. In control provinces, the DTP-3 coverage rate increased from 54.6% to 71.9% (OR 2.1, 95% CI 1.3–3.5). Stockouts reduced from 79% to less than 1%. |
| Kaewkungwal et al (2010)^46^ | n=4,200 children | Thailand | pilot study (cohort) | MCCM app for appointment reminders | On-time vaccination rate | Compared to the control group, the OR of being on time for vaccination were 2.13 (95% CI 1.79-2.52) |
| Dolan et al (2022)^47^ | n= 507,571 children | Tanzania | quasi-experimental study | Electronic immunisation registry | On-time vaccination rate | For DTP-1 vaccinations, the on-time vaccination rate increased by 5% monthly (OR: 1.05, 95% CI: 1.05-1.06). For DTP-2 and DTP-3, it increased by 2% (OR: 1.02, 95% CI: 1.01-1.03; and OR: 1.01, 95% CI: 1.00-1.03, respectively). However, for MCV-1, the rate decreased by 6% per month (OR: 0.94, 95% CI: 0.92-0.97) |
| Yunusa et al (2022)^48^ | n=541 children | Nigeria | quasi-experimental study | SMS text reminders + follow up calls | Penta-3 coverage rate | Completion rates for the three doses of the pentavalent vaccine were observed to be higher for children in the reminder group (59.4%) compared to those in the control group (34.1%). |
| Nguyen et al (2017)^49^ | NA | Vietnam | quasi-experimental study | Electronic immunisation registry | Penta-3 and MCV-1 coverage rate Penta-3 and MCV-1 dropout rate Full coverage rate at 12 months of age | The timeliness of Penta-3 and MCV-1 vaccinations improved from 53.6% to 77.2%, and from 70.4% to 92.3%, respectively. Dropout rates for Penta and MCV-1 declined from 4.2% to 0% and 12.8% to 0%, respectively. Additionally, full immunisation coverage for children under one year old rose significantly from 75.4% to 99.2% |
| El-Halabi et al (2023)^50^ | n=936 children | Jordan | quasi-experimental study | CIMA-App | On-time vaccination rate Default Rate | In the intervention group, 24.6% came back on time, versus 20.7% in the control group (p = 0.01). Defaulters were 22.5% and 27.8% in the intervention and control groups, respectively. Kaplan Meier survival analysis showed a statistically significant reduction in coming back, within 0–14 days, within the vaccine appointment period (p < 0.01) |
| Ramanujapuram, (2014)^51^ | NA | India | quasi-experimental study | “Bulletin Board” that digitally captures needs (demand) and availability (supply) of goods in real-time from any location using mobile phones, and broadcasts this information to vendors and managers, upstream in the supply chain | Stock availability | vaccine stock availability increases to 99% and replenishment responsiveness improve by 64%. |
| Jalloh et al (2020)^52^ | n=10 Health facilities | Sierra Leone | Mixed Method study | Electronic immunisation registry | Completeness between electronic and paper-based records | VaxTrac captured < 65% of the vaccine doses reported in the paper-based sources |
| Siddiqi et al (2023)^53^ | n=4,613 children | Pakistan and Bangladesh | Mixed Method study | Immunisation decision support system (iDSS) | Accuracy in constructing age-appropriate vaccination schedules as per the WHO recommended EPI guidelines. | The iDSS correctly scheduled 99.8% of all age-appropriate immunisation doses compared with the gold standard (96.8%). |
| Oladepo et al (2021)^54^ | n = 3,500 children | Nigeria | quasi-experimental study | SMS text reminders | On-time vaccination rate | Adherence to routine immunisation schedules and completion rates were higher in the Intervention group (76.0%) than in the control group (73.3%), p>0.05. Intervention group showed significantly higher completion rates for measles and yellow fever vaccines at 55.3% and 75.9%, respectively, compared to 26.8% and 23.9% in the control group |
| Negandhi et al (2016)^55^ | Health facilities (n=172)  District vaccine stores (n=38)  Regional vaccine stores (n=7) | India | Mixed method study | Mobile-based Effective Vaccine Management (EVM) system | Cold chain capacity EVM performance score | Cold chain capacity expanded from 49% to 87%. Significant improvements were noted in overall EVM criteria indicators across health facilities, district vaccine stores, and regional vaccine stores, increasing to 82%, 84%, and 80% respectively, from previous levels of 51%, 46%, and 43%. Similarly, EVM category indicators rose to 83%, 84%, and 76% respectively, compared to the earlier 54%, 53%, and 54% |
| Chan et al (2017)^56^ | n=1,273 children | Mongolia | cross sectional | Electronic immunisation registry | Accuracy and completeness between electronic and paper-based records | Completeness (90.9%; 95% CI: 88.4-93.4) and accuracy (93.3%; 95% CI: 84.1-97.4) of the electronic immunisation register were high when compared to written records. The increase in completeness over time indicated a delay in data entry |
