# Supplementary material for: The impact of information and communication technology on immunisation and immunisation programmes in low-income and middle-income countries: a systematic review and meta-analysis
Source: eBioMedicine. 2024 Dec 21;111:105520. doi: 10.1016/j.ebiom.2024.105520 (PMC11732194; doi:10.1016/j.ebiom.2024.105520)
Supplement: Supplementary File 5 [file mmc5.docx]

Supplementary file no 5: description of the digital health interventions

| Kawakatsu et al (2020)^30^ | 1.Personal information of the child was documented in the database via a mobile app, linking individual details to the system.  2. A QR code was generated for each child, and subsequent appointments were scheduled through the app.  3. Customized SMS text reminders in English were sent two days prior to the appointment, and follow-up reminders were dispatched 7 days after a missed appointment. |
| --- | --- |
| Bangure et al (2015)^31^ | Caregivers received routine health education and automated messages for appointments at 6, 10, and 14 weeks. SMS reminders were sent 7, 3, and 1 day prior to the scheduled appointments. |
| Kagucia et al (2021)^32^ | 1. The caregivers received SMS reminders two times: 3 days and one day prior to the scheduled appointments  3. The reminder before the appointment was the same across intervention arm and consisted of reminders + motivational phrases  in Dholuo, Kiswahili or English languages. |
| Tsafack et al (2015)^33^ | Caregivers were given the investigation team's phone contact and instructed to send a mobile phone signal or “beep” to notify of any medical events occurring within 30 days post-immunization. |
| Seth et al (2018)^34^ | An encrypted, cloud-based software platform developed by Royal Datamatics Pvt Ltd (New Delhi, India) was used for record keeping and delivery of automated mobile phone reminders and compliance linked incentives. |
| Gibson et al (2017)^35^ | 1.Villages were randomly and evenly allocated to four groups: control, SMS only, SMS plus 75 Kenya Shilling (KES) incentive, and SMS plus 200 KES (85 KES = USD$1).  2. The RapidSMS server is utilized by healthcare workers to dispatch birth notifications via text messages. This system efficiently identifies eligible caregivers and their infants, enabling the sending of SMS reminders for essential follow-up and care. |
| Dissieka et al (2019)^36^ | Caregivers received SMS or voice message reminders, based on their preference. One reminder before each scheduled facility visit and two additional reminders in the event of non-attendance. |
| Haji et al (2016)^37^ | Caregivers received SMS reminders from an automated web-based system two days before and on the day of the scheduled due dates for the second and third doses of the pentavalent vaccine.  The text messages were sent in Kiswahili and English. |
| Ateudjieu et al (2014)^38^ | 1.Mobile numbers of AEFI focal points at health facilities in the SMS group were verified by calling each owner. The messages were recorded, cross-checked, and saved onto a mobile phone.  2.Over four consecutive weeks, standardized SMS messages were sent every Monday at 8:00 a.m. in one language (French or English) and on Tuesday in the other language, to all AEFI focal points in the SMS group.  3.The message content, consistent each week, included a reminder about the MenAfriVacT AEFI surveillance period.  4.The mobile phone's “delivery report” feature was utilized to confirm receipt and opening of the messages. |
| Ekhaguere et al (2019)^39^ | 1. A customized Windows software application was designed to send automated voice call text and email immunization reminders  2. A secure cloud communications platform, called Twilio, was Integrated into the app. Messaging and voice were sent by Twilio through the app.  3. The immunization reminders were auto calculated from date of birth of the child and tailored to the local immunization schedule  4. Reminders were sent 2 days and the day before the scheduled date of the Penta-1,2, 3 and measles immunizations. |
| Mekonnen et al (2021)^40^ | Routine vaccination reminders and additional mobile text messages were sent one day prior to the scheduled vaccination visits at the sixth week, tenth week, fourteenth week, and ninth month post-childbirth. |
| Brown et al (2016)^41^ | 1. Intervention arm A: cellphone calls for reminder/recall intervention sessions were implemented.  2. Intervention arm B: two days refresher training on theory and practice of immunization was conducted for Primary Health Care immunization providers  3. Telephone reminder intervention involved one session of two cell phone reminder calls made to either a parent of a child or any contact person whose cell phone number has been recorded in the study by the mother. Reminders were sent two days and a day before the immunization appointment. |
| Eze at al (2015)^42^ | 1. Reminder messages were dispatched a day before appointments, and recall messages were sent one day before the next immunization session whenever babies missed their scheduled appointments  2. All text messages were sent through an internet-based web-to-SMS (Bulk SMS) service and tagged with the client’s health facility name for easy recognition. |
| Domek et al (2019)^43^ | 1. SMS reminders were dispatched three, two, and one day prior to the scheduled dates for visits 2 and 3.  2. The local data manager entered each child’s name, scheduled visit date, clinic, and caregiver’s mobile number into a computer system connected to the SMS server both at enrollment and after visit.  3. Automated SMS texts were generated using a customized computer-based software. |
| Kazi et al (2018)^44^ | 1.In addition to standard counseling, caregivers received four SMS reminders in the week when the enrolled child was due for EPI vaccines as per the Routine Immunization schedule.  2.The same message was dispatched when the child reached 6, 10, and 14 weeks of age. |
| Prosser et al (2017)^45^ | 1.HERMES (Highly Extensible Resource for Modeling Supply Chains) is a simulation tool utilized in the study to assess the effects of three alternative supply chain designs on product availability and logistics costs.  2.HERMES generates detailed discrete-event simulation models for comparing efficiencies of different system designs.  3.The EVM assessment was employed to evaluate the impact of system redesign in the Come district of Benin. |
| Kaewkungwal et al (2010)^46^ | 1.Data on maternal care and child immunization from Thailand's Healthcare Information System (HCIS) is transferred to the Mobile-based Community Case Management (MCCM) module.  2.The program then creates appointment dates for each client and dispatches SMS reminders to healthcare workers for follow-up visits.  3.An SMS is automatically sent directly to clients' personal cell phones a few days before their scheduled appointment. |
| Dolan et al (2022)^47^ | Set up of the Tanzania Immunization Registry (TImR), with online and offline functionality that enabled automated, simplified reports; development of logistics management information systems; provision of targeted supportive supervision for HCWs. |
| Yunusa et al (2022)^48^ | 1.The SMS reminders were sent to caregivers three days prior to the due date of immunization and on the scheduled day.  2. Tracking of the immunization status of the children started in the health facility immediately after sending the first SMS reminders and continued for three months after the last SMS. |
| Nguyen et al (2017)^49^ | 1.The data for this study, sourced from ImmReg (a locally developed custom software program), encompassed all registered births  2.Full immunization rate, dropout rate, and vaccination timeliness were assessed before and after the intervention.  3.Furthermore, a rapid survey assessed parental willingness to pay for immunization reminder SMS. |
| El-Halabi et al (2023)^50^ | Caregivers downloaded the CIMA app, at no cost, on their personal devices (Android only) with the help of the study staff. The CIMA app included four layers: (i) Health promotion messages for the benefits of vaccination (ii) Storing the post of vaccination for each child, according to the vaccination schedule (iii) Displaying the vaccination schedule, for each child, using green, orange and red colors depending on vaccination status if it was received, due or overdue respectively; (iv) Appointment reminder was displayed on the user’s phones at four different time-points prior the vaccination schedule (one week, three days, 1 day and the morning of the appointment). Then the users received two notifications in the coming days of the scheduled vaccine in case of missing the appointment. |
| Ramanujapuram (2016)^51^ | To address the problem of stock availability, a “Bulletin Board” digitally captures needs (demand) and availability (supply) of goods in real-time from any location using low-end mobile phones, and broadcasts this information to vendors and managers, upstream in the supply chain. |
| Jalloh et al (2020)^52^ | Records from the under-two register were manually entered into the VaxTrac system, assigned a unique identification number, and linked to a QR barcode. This barcode, affixed to the under-two register, was used for real-time tracking during vaccination sessions. |
| Siddiqi et al (2023)^53^ | 1.The intervention, iDSS, is for mobile use. iDSS is a mobile software which autonomously updates a child's immunization schedule after each visit, adjusting for missed or delayed vaccinations based on birth date and prior vaccines, eliminating manual calculations by vaccinators. It incorporates changes in the EPI schedule and new vaccines into its algorithm.  2.The iDSS displays current and upcoming vaccines through a color-coded interface for easy interpretation. It is packaged as an application programming interface, functioning both independently and interoperable with other platforms like web-based or mobile-based electronic immunization registries.  3.The system operates in a 2-step process with a data entry form and a display interface showing the vaccination schedule. |
| Oladepo et al (2020)^54^ | 1.The reminder text messages were sent at scheduled intervals based on focus group discussions (three times a week between 7.00 and 7.15 am). |
| Negandhi et al (2016)^55^ | The mHealth Effective Vaccine Management (EVM) tool digitally assess the current state of vaccine cold chain management systems. This process enhances the efficiency of vaccine supply chain evaluation, replacing the traditional practice of using paper forms for assessments. |
| Chan et al (2017)^56^ | Immunization nurses recorded the following details in a registration book: name, national identification number, age, address, phone number, and vaccination date. This data was subsequently entered into a web-based electronic immunization registry. The system allowed real-time access to information from the electronic registers. |
