# Supplementary material for: The impact of information and communication technology on immunisation and immunisation programmes in low-income and middle-income countries: a systematic review and meta-analysis
Source: eBioMedicine. 2024 Dec 21;111:105520. doi: 10.1016/j.ebiom.2024.105520 (PMC11732194; doi:10.1016/j.ebiom.2024.105520)
Supplement: Supplementary File 6 [file mmc6.docx]

Supplementary file 6: Risk of bias assessment

**RCT (Joanna Briggs Institute Critical appraisal tools 2023)**

| **Study** | **Randomization** | **Concealed allocation** | **Similar baseline** | **Participants blind to assignment** | **Treatment delivers blind** | **Outcomes assessiors blind** | **Groups treated identically** | **Follow up complete** | **Intention-to-treat analysis** | **Outcomes measured in the same way** | **Outcomes measured in a reliable way** | **Proper analysis** | **Proper study design** | **QUALITY** |
| --- | --- | --- | --- | --- | --- | --- | --- | --- | --- | --- | --- | --- | --- | --- |
| Ateudjieu et al (2014)^38^ | 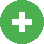 | 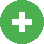 | 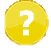 | **NA** | 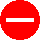 | 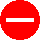 | 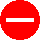 | 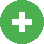 | 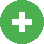 | 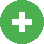 | 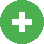 | 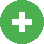 | 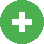 | **Medium** |
| Bangure et al (2015)^31^ | 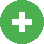 | 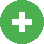 | 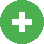 | **NA** | 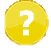 | 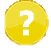 | 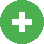 | 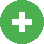 | 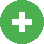 | 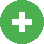 | 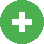 | 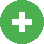 | 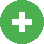 | **High** |
| Brown et al (2016)^41^ | 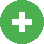 | 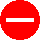 | 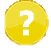 | **NA** | 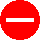 | 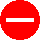 | 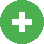 | 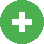 | 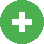 | 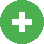 | 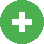 | 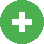 | 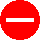 | **Low** |
| Haji et al (2016)^37^ | 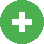 | 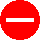 | 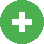 | **NA** | 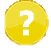 | 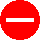 | 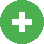 | 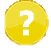 | 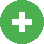 | 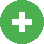 | 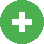 | 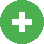 | 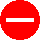 | **Low** |
| Eze at al (2015)^42^ | 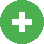 | 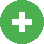 | 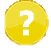 | **NA** | 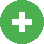 | 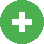 | 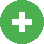 | 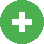 | 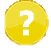 | 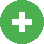 | 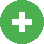 | 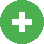 | 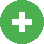 | **High** |
| Tsafack et al (2015)^33^ | 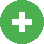 | 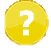 | 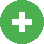 | **NA** | 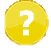 | 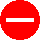 | 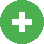 | 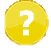 | 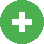 | 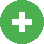 | 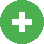 | 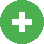 | 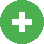 | **Medium** |
| Gibson et al (2017)^35^ | 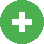 | 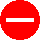 | 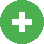 | **NA** | 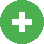 | 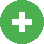 | 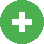 | 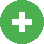 | 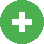 | 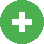 | 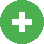 | 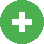 | 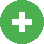 | **High** |
| Kazi (2018)^44^ | 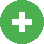 | 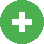 | 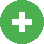 | **NA** | 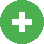 | 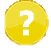 | 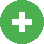 | 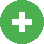 | 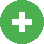 | 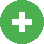 | 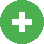 | 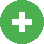 | 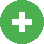 | **High** |
| Seth et al (2018)^34^ | 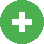 | 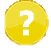 | 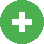 | **NA** | 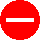 |  |  |  |  |  |  |  |  | **Medium** |
| Ekhaguere et al (2019)^39^ |  |  |  | **NA** |  |  |  |  |  |  |  |  |  | **High** |
| Dissieka et al (2019)^36^ |  |  |  | **NA** |  |  |  |  |  |  |  |  |  | **Medium** |
| Domek et al (2019)^43^ |  |  |  | **NA** |  |  |  |  |  |  |  |  |  | **High** |
| Kawakatsu et al (2020)^30^ |  |  |  | **NA** |  |  |  |  |  |  |  |  |  | **Medium** |
| Kagucia et al (2021)^32^ |  |  |  | **NA** |  |  |  |  |  |  |  |  |  | **High** |
| Mekonnen et al (2021)^40^ |  |  |  | **NA** |  |  |  |  |  |  |  |  |  | **High** |

**Quasi-Experimental studies (**Joanna Briggs Institute Critical appraisal tools 2023)

| **Study** | **Temporal relationship correct** | **Differences between participants** | **Difference in care received** | **Indipendent control group** | **Multiple measurements of the outcome** | **Loss to follow** | **Outcomes measured in the same way** | **Outcomes measured in a reliable way** | **Appropriate analysis** | **QUALITY** |
| --- | --- | --- | --- | --- | --- | --- | --- | --- | --- | --- |
| Kaewkungwal et al (2010)^46^ |  |  |  |  |  |  |  |  |  | **Medium** |
| Dolan et al (2022)^47^ |  |  |  |  |  |  |  |  |  | **High** |
| Yunusa et al (2022)^48^ |  |  |  |  |  |  |  |  |  | **High** |
| Nguyen et al (2017)^49^ |  |  |  |  |  |  |  |  |  | **Medium** |
| El-Halabi et al (2023)^50^ |  |  |  |  |  |  |  |  |  | **High** |
| Prosser et al (2017)^45^ |  |  |  |  |  |  |  |  |  | **Medium** |
| Ramanujapuram (2014)^51^ |  |  |  |  |  |  |  |  |  | **Low** |

**Cross-sectional studies (**Joanna Briggs Institute Critical appraisal tools 2023)

| **Study** | Chan et al (2017)^56^ |
| --- | --- |
| **1.research question clear** |  |
| **2.Study population specified and defined** |  |
| **3.Was the participation rate of eligible persons at least 50%?** |  |
| **4.Were all the subjects selected from the same populations (including the same time period)? Were inclusion and exclusion criteria for being in the study prespecified and applied uniformly to all participants?** |  |
| **5. Was a sample size justification, power description, or variance and effect estimates provided?** |  |
| **6.For the analyses in this paper, were the exposure(s) of interest measured prior to the outcome(s) being measured?** |  |
| **7.Was the timeframe sufficient so that one could reasonably expect to see an association between exposure and outcome if it existed?** |  |
| **8.For exposures that can vary in amount or level, did the study examine different levels of the exposure as related to the outcome (e.g., categories of exposure, or exposure measured as continuous variable)?** |  |
| **9.Were the exposure measures (independent variables) clearly defined, valid, reliable, and implemented consistently across all study participants?** |  |
| **10.Was the exposure(s) assessed more than once over time?** |  |
| **11.Were the outcome measures (dependent variables) clearly defined, valid, reliable, and implemented consistently across all study participants?** |  |
| **12.Were the outcome assessors blinded to the exposure status of participants?** |  |
| **13.Was loss to follow-up after baseline 20% or less?** |  |
| **14.Were key potential confounding variables measured and adjusted statistically for their impact on the relationship between exposure(s) and outcome(s)?** |  |
| **QUALITY** | **High** |

**Mixed-Methods studies (**Mixed methods appraisal tool, 2018 version)

|  | **Question** | Jalloh et al (2020)^52^ | Negandhi et al (2016)^55^ | Siddiqi et al (2023)^53^ | Oladepo et al (2021)^54^ |
| --- | --- | --- | --- | --- | --- |
| **Screening questions** | **S1 Clear research question** |  |  |  |  |
|  | **S2 Proper data to answer the research question** |  |  |  |  |
| **1. Qualitative** | **1.1 Appropriate qualitative approach** |  |  |  |  |
|  | **1.2 Appropriate qualitative data collection** |  |  |  |  |
|  | **1.3 Finding adequately derived from the data** |  |  |  |  |
|  | **1.4 Adequate data interpretation** |  |  |  |  |
|  | **1.5 Coherence between data sources, analysis and interpretation** |  |  |  |  |
| **3. Quantitative non-randomized** | **3.1 Participants rapresentative of target population** |  |  |  |  |
|  | **3.2 Proper assessment of exposure and outcome** |  |  |  |  |
|  | **3.3 Complete outcome data** |  |  |  |  |
|  | **3.4 Confounders accounted in the study design and analysis** |  |  |  |  |
|  | **3.5 Intervention administered as intended** |  |  |  |  |
| **5. Mixed Methods** | **5.1 Mixed method design appropriate for the research question** |  |  |  |  |
|  | **5.2 Different components of the study properly integrated** |  |  |  |  |
|  | **5.3 Output of the integration properly interpreted** |  |  |  |  |
|  | **5.4 Divergences and inconsistences addressed** |  |  |  |  |
|  | **5.5 Quality criteria of data collection respected** |  |  |  |  |
| **QUALITY** |  | **High** | **Low** | **High** | **High** |
